# mRNA-dendritic cell antigen vaccine potentiates CD38 CAR-T Cell therapy for relapsed/refractory acute myeloid leukemia

**DOI:** 10.7150/thno.139339

**Published:** 2026-08-24

**Authors:** Yu Lu, Xin Cai, Yanhui Li, Yuyuan Lv, Yutong Tang, Yuanyuan Li, Jiazi Zhou, Ailin Zhang, Pengchen Gu, Ziyi Hao, Qingya Cui, Jianhong Chu, Depei Wu, Xiaowen Tang

**Affiliations:** 1 National Clinical Research Center for Hematologic Diseases, Jiangsu Institute of Hematology, The First Affiliated Hospital of Soochow University, Suzhou, 215006, China.; 2 Institute of Blood and Marrow Transplantation, Collaborative Innovation Center of Hematology, Suzhou Medical College, Soochow University, Suzhou, 215123, China.; 3 CSPC Pharmaceutical Group Co., Ltd., 896 East Zhongshan Road, Shijiazhuang 050035, China.

**Keywords:** chimeric antigen receptor T cell, acute myeloid leukemia, CD38, lipid nanoparticle, mRNA vaccine, antigen-presenting cell

## Abstract

**Background:**

Chimeric antigen receptor (CAR) T-cell therapy has emerged as a promising strategy for management of relapsed/refractory acute myeloid leukemia (R/R AML). Safety and preliminary activity of CD38-targeted CAR-T cells in R/R AML were demonstrated in our prior study; however, relapse with CD38-positive blasts persisted, which was partly associated with insufficient effector function and persistence of CAR-T cells. Given that CAR-T cell hypofunction is promoted by inadequate antigen stimulation, a lipid nanoparticle-delivered CD38 mRNA vaccine was developed to enhance CD38 CAR-T cell efficacy via exogenous antigen presentation.

**Methods:**

The CD38 mRNA-LNP vaccine was prepared, and its biodistribution profile and tolerability were evaluated in BALB/c mice. The capacity of this vaccine to augment the activation, proliferative capacity, memory subset distribution, cytokine secretion, and antileukemic efficacy of CD38-knockout CAR-T cells (Ko-38CAR) was systematically assessed *in vitro*. Transcriptomic and ATAC-seq analyses were employed to delineate the underlying molecular regulatory networks. *In vivo* antitumor efficacy was validated in a THP-1 AML xenograft model.

**Results:**

The vaccine (107 nm, 91.67% encapsulation) exhibited splenic APC tropism and induced pulsatile CD38 expression that peaked at 24 h and declined markedly by 48 h. Activation, proliferation, cytotoxicity, and memory differentiation of Ko-38CAR cells were dose-dependently augmented by the vaccine. Furthermore, the vaccine promoted the activation of polyamine metabolism (e.g., *ODC1*), *JAK-STAT* and* MAPK* signaling pathways, upregulated *BCL2* expression, and enhanced chromatin accessibility of cell cycle and effector genes in CAR-T cells. *In vivo*, vaccine administration promoted CAR-T cell expansion, improved tumor control, and prolonged survival.

**Conclusions:**

CD38 mRNA-LNP vaccine can efficiently deliver pulsatile CD38 antigen to splenic APCs and augment the proliferation, effector function, and persistence of CD38 CAR-T cells through dual CAR and costimulatory signaling, which may represent a promising strategy to overcome CAR-T cell dysfunction in R/R AML.

## Introduction

Adoptive immunotherapy mediated by genetically engineered T lymphocytes expressing chimeric antigen receptors has achieved breakthrough progress and emerged as a transformative modality in the field of B-cell malignancies 1. However, its efficacy in malignancies such as acute myeloid leukemia (AML) remains suboptimal, limiting broader clinical application of this strategy. The *in vivo* proliferative capacity and persistence of CAR-T cells are critical determinants of therapeutic efficacy, and accumulating evidence indicates that restricted expansion and inadequate persistence constitute the principal causes of non-response and early relapse 2, 3.

Results from our previous clinical study (NCT04351022) demonstrated that CD38-targeted CAR-T cells with the anti-CD38 scFv derived from daratumumab achieved complete remission or complete remission with incomplete hematologic recovery in 66.7% (4/6) of patients with AML relapsing after allogeneic hematopoietic stem cell transplantation, with a median remission duration of 191 days (range: 117–261 days). These findings provided preliminary evidence for the efficacy and safety of CD38-targeted CAR-T therapy in R/R AML 4. Nevertheless, the clinical efficacy of CAR-T cell therapy in R/R AML remains substantially inferior to that achieved in B-cell malignancies, which is primarily attributable to suboptimal overall response rates and limited response durability 3, 5. Emerging evidence indicates that the level of antigen exposure is a critical determinant of CAR-T cell activation and proliferative capacity. Infusion of antigen-loaded cells as an interventional strategy can reinvigorate CAR-T cell-mediated immune surveillance against residual leukemic blasts, thereby improving therapeutic durability 6. Furthermore, a study in solid tumors demonstrated that presentation of target antigens by antigen-presenting cells effectively promotes selective clonal expansion of CAR-T cells 7, 8. Additionally, Mohammad Rashidian and colleagues developed a CAR enhancer comprising a BCMA antigen fused with low-affinity interleukin-2 which selectively recognizes and binds CAR-T cells, thereby coordinately regulating their expansion, antitumor effector function, and differentiation into memory phenotypes 9. Collectively, given the numerous challenges confronting CAR-T therapy in R/R AML, antigen-boosting strategies represent a feasible and promising interventional approach.

In this study, to overcome the dual challenges of limited response rates and short-lived therapeutic efficacy associated with CD38 CAR-T therapy in R/R AML, a lipid nanoparticle-based CD38 mRNA-dendritic cell antigen vaccine (designated the 38-mRNA vaccine) was designed. Capitalizing on the inherent tropism of lipid nanoparticles for antigen-presenting cells, efficient expression of the CD38 antigen on splenic APCs was enabled by this vaccine. Through this strategy, the *in vivo* activation, memory formation, and proliferative potential of CD38 CAR-T cells were enhanced, and their antitumor effector function was markedly potentiated, without eliciting significant safety concerns. In a CDX model of R/R AML, the survival of tumor-bearing mice was significantly prolonged by the combination therapy. Collectively, the superiority and translational potential of CD38 CAR-T cells combined with the 38-mRNA vaccine as an emerging therapeutic strategy for R/R AML are reported for the first time in this study.

## Methods

### Cell lines and culture

All human cell lines were purchased from the American Type Culture Collection (ATCC), with the exception of THP-1-luc, which was obtained from IMMOCELL (Xiamen, Fujian, China). The human relapsed/refractory acute myeloid leukemia cell lines MOLM-13, THP-1-luc, and THP-1 were maintained in RPMI 1640 medium supplemented with 10% fetal bovine serum (10099141C, GIBCO).

### Human peripheral blood mononuclear cells (PBMCs) and dendritic cells (DCs)

All human peripheral blood samples were collected from healthy donors following the principles of the Declaration of Helsinki. Written informed consent was obtained from all participants, and the study protocol was approved by the Ethics Committee of the First Affiliated Hospital of Soochow University (Suzhou, China). Peripheral blood mononuclear cells (PBMCs) were isolated from fresh anticoagulated whole blood by density gradient centrifugation using Human lymphocyte separation medium (7111011, Dakewe). Monocytes were subsequently enriched from PBMCs by positive selection using human anti-CD14 microbeads (Miltenyi Biotec) according to the manufacturer’s instructions. To generate immature dendritic cells (iDCs), the enriched monocytes were cultured for 5 days in differentiation medium consisting of RPMI 1640 GlutaMAX™ supplemented with 50 IU/mL penicillin, 50 μg/mL streptomycin, 1 mM sodium pyruvate, non-essential amino acids, and 10% fetal bovine serum 10099141C, GIBCO), together with 1,000 IU/mL recombinant human GM-CSF (91113ES60; Yeasen, Shanghai, China) and 1,000 IU/mL recombinant human IL-4 (Y02201, EastMabBio).

### Preparation of CD38-mRNA Vaccine lipid nanoparticles (38-mRNA Vaccine) and transfection of dendritic cells and macrophages

The luciferase-mRNA vaccine, CD38-mRNA vaccine, and empty control vaccine were synthesized by CSPC Pharmaceutical Group Co., Ltd. As previously described 10 and in Patent No. WO2024245341A1, RNA encoding the CD38 antigen or luciferase was produced by *in vitro* transcription from DNA plasmid templates that were optimized to enhance RNA stability and translational efficiency. For the preparation of mRNA-LNPs, four lipids—SM-102, mPEG-DMG-2K, DSPC, and cholesterol—were dissolved in anhydrous ethanol at a molar ratio of 50%: 1.5%: 10%: 38.5% to form the lipid phase. Separately, mRNA was diluted in a citrate-sodium chloride buffer (pH 4.0) to obtain the aqueous phase. The lipid phase and aqueous phase were then mixed at a volume ratio of 1:3 using a T-mixer, allowing the lipids to encapsulate the mRNA and form lipid nanoparticles. Subsequently, the nanoparticle suspension was subjected to a two-step tangential flow filtration (TFF) process for buffer exchange and removal of ethanol: first dialyzed against citrate buffer (pH 4.0), and then against Tris-acetate-sucrose buffer (pH 7.4). After the TFF steps, the final product was sterilized by terminal filtration to obtain uniform and stable mRNA vaccine lipid nanoparticles.

For dendritic cell and macrophages transfection, induced mature donor-matched dendritic cells and macrophages were plated in 12-well plates at equal cell numbers according to previously reported protocols 8. Different doses of the 38-mRNA vaccine were added according to the experimental design, and cells were incubated for 20 hours before termination of transfection. Subsequent experiments were performed according to the specific experimental protocols 8.

### Flow cytometry

Staining procedures were performed according to previously reported protocols. Data were acquired using a BD Celesta flow cytometer running FACS Diva software and analyzed with FlowJo v10.1 software. Antibodies and staining reagents used in flow cytometry are summarized below.

The following antibodies were purchased from BioLegend: APC-Cy7 rat anti-mouse CD45, PE-CF594 rat anti-mouse CD19, PE rat anti-mouse F4/80, FITC hamster anti-mouse TCR β chain, BV421 rat anti-mouse CD49b, BV605 mouse anti-human CD38, APC hamster anti-mouse CD11c, PerCP-Cy5.5 rat anti-mouse CD11b, PE rat anti-mouse I-A/I-E, APC mouse anti-human CD38, propidium iodide (PI), BV510 fixable viability dye, APC-Cy7 mouse anti-human CD4, PerCP-Cy5.5 mouse anti-human CD8, PE mouse anti-human CCR7, BV650 mouse anti-human CD45RA, PE mouse anti-human CD25, APC mouse anti-human CD69, APC mouse anti-human CD3, BV785 mouse anti-human CD45, and anti-mouse Fc receptor blocker.

### Statistical analysis

The sample size was determined based on previously published literature. Statistical significance was assessed using unpaired two-tailed Student's t-test, one-way ANOVA, or two-way ANOVA as indicated in the figure legends. For multiple comparisons, ANOVA was followed by Tukey's post hoc test or Bonferroni correction. Survival curves were constructed using the Kaplan-Meier method, and survival differences between groups were compared using the log-rank (Mantel-Cox) test. A P value or adjusted *P* value < 0.05 was considered statistically significant. All statistical analyses and graphical representations were performed using GraphPad Prism software v8.0.

## Results

### Preparation and biodistribution of the 38-mRNA vaccine

Using an optimized SM102 formulation 10, we successfully prepared lipid nanoparticles encapsulating CD38 protein-encoding mRNA and luciferase mRNA. Physicochemical characterization revealed that the key parameters of the 38-mRNA vaccine and luciferase-mRNA vaccine were highly consistent: mean particle sizes were 107 nm and 103 nm, mean pH values were 7.43 and 7.40, polydispersity indices were 0.043 and 0.040, and encapsulation efficiencies reached 91.67% and 92.00%, respectively (Figure 1A-B). Cryo-electron microscopy showed that both vaccines exhibited uniformly sized spherical morphology (Figure 1C).

The *in vivo* biodistribution of the mRNA nanoparticle vaccine was assessed using luciferase-mRNA lipid nanoparticles. The results demonstrated that the vaccine predominantly distributed to the liver and spleen, with a slight increase observed in the bone marrow, and the most pronounced bioluminescence signal was detected in the splenic region. At 48 h post-administration, signal intensities across all tissues exhibited marked attenuation (Figure 1D-G).

Furthermore, to evaluate the tissue distribution pattern and potential safety of the 38-mRNA vaccine, we administered an equivalent dose (20 μg per mouse) of the 38-mRNA nanoparticle vaccine via tail vein injection and performed comprehensive assessment using immunohistochemical staining, H&E staining, and TUNEL assay (Figure S1A). The results showed that CD38 protein expression was predominantly localized within the spleen and liver tissues. Concurrently, no evident morphological abnormalities or increased apoptosis were observed in any major organs examined, suggesting that the vaccine possesses favorable *in vivo* safety (Figure S1B-D).

Based on the marked splenic and bone marrow tropism of the mRNA nanoparticle vaccine revealed by *in vivo* imaging, we further characterized the target cell types of the 38-mRNA vaccine within the splenic and bone marrow microenvironments by flow cytometry. With reference to previous literature 8, 11, target cells were identified as follows: B cells (CD45⁺CD19⁺), T cells (CD45⁺TCRβ⁺), NK cells (CD45⁺CD49b⁺), macrophages (F4/80⁺), and dendritic cells (CD45⁺CD11c⁺CD11b⁻MHC II⁺ [I-A/I-E⁺]). Following tail vein injection of 20 μg of the 38-mRNA nanoparticle vaccine, flow cytometric analysis of the spleen revealed that CD38 antigen presentation occurred predominantly in antigen-presenting cells, including macrophages and dendritic cells, with mean positivity rates of 42.62 ± 6.1% and 43.92 ± 10.99% at 24 h, respectively. In contrast, CD38 expression was barely detectable in B cells, T cells, and NK cells. By 48 h post-administration, the positivity rates of macrophages and dendritic cells had declined significantly to 14.20 ± 3.81% and 15.21 ± 4.25%, respectively (Figure 2A-B, D). This finding indicates that vaccine-elicited CD38 expression exhibited pulsatile kinetics rather than persistent presentation, an expression modality that mitigates CAR-T cell functional exhaustion caused by chronic, sustained antigenic stimulation. Compared with those in the spleen, CD38 expression levels across all immune cell subsets in the bone marrow remained at a lower baseline, with positivity rates below 10% (Figure 2C, E).

### The 38-mRNA vaccine enhances the function and antitumor efficacy of CD38-targeted CAR-T cells

Based on the tropism of the 38-mRNA vaccine for dendritic cells revealed in prior *in vivo* experiments, an *in vitro* dendritic cell culture system was established to systematically evaluate the transfection efficiency, *in vitro* safety, and potentiating effects on CD38 CAR-T cells of the vaccine. To determine the transfection efficiency and potential cytotoxicity of the 38-mRNA vaccine in dendritic cells, *in vitro* differentiated dendritic cells were incubated with varying concentrations of the vaccine for 20 h, after which CD38 surface expression and cell viability were quantified by flow cytometry to establish the optimal working concentration for subsequent functional assays (Figure 3A). The results demonstrated that CD38 expression on the surface of dendritic cells increased in a dose-dependent manner with escalating vaccine doses (mean ± SD MFI: 0 μg, 62.8 ± 3.9; 0.1 μg, 1365 ± 392; 0.5 μg, 4953 ± 1014; 5 μg, 44,120 ± 8049), and cell viability remained above 90% at both the 0.1 μg and 0.5 μg doses (Figure 3B-D). Furthermore, the expression of the costimulatory molecule CD86 on the surface of dendritic cells was enhanced in a dose-dependent manner, while CD40 expression showed an upward trend without statistical significance (Figure 3E-H).CD38-targeted CAR-T cells were constructed using the single-chain variable fragment derived from daratumumab. Given the adverse impact of high endogenous CD38 expression on CAR-T cell expansion and previous reports indicating that CD38 knockout can enhance CAR-T cell function through metabolic reprogramming 12, 13, we employed the CRISPR-Cas9 system to knock out the CD38 gene in CD38 CAR-T cells. The results showed that the CAR positivity rate of Ko-38CAR cells on day 14 (mean ± SD, 64.9 ± 8.4%) was lower than that of 38CAR cells (mean ± SD, 98.5% ± 0.30%), although both groups exhibited increased positivity compared with day 7 (Figure 4A–C). CD38 expression analysis revealed that the non-transduced control group (NT group) maintained high CD38 expression (mean 93.0%), whereas CD38 expression in both the 38CAR and Ko-38CAR groups was effectively suppressed to near-zero levels (Figure 4D–E). Notably, the 38CAR group exhibited a pronounced expansion defect, with expansion folds substantially lower than those of the NT group. In contrast, the expansion capacity of the Ko-38CAR group was substantially restored and did not differ significantly from that of the NT group (Figure 4F).

Following the experimental system reported in previous literature 8, we further evaluated the impact of 38-mRNA nanoparticle vaccine at different dosages on CD38 CAR-T cell function. Dendritic cells were pretreated with 0 μg, 0.1 μg, or 0.5 μg of the 38-mRNA nanoparticle vaccine for 20 hours and subsequently co-cultured with CTV-labeled Ko-38CAR cells. The activation status and memory differentiation phenotype CAR-T cells were assessed after 24 hours of co-culture, and proliferation kinetics was analyzed after 96 hours (Figure 4G). Flow cytometric analysis revealed that the mean proliferation rate of Ko-38CAR cells cultured alone was 5.52 ± 0.73%, which increased to 35.87 ± 10.45% upon co-culture with untreated dendritic cells (Ko-38CAR+DC+0 μg). Furthermore, the proliferation rate rose to 48.73 ± 9.20% in the 0.1 μg vaccine-treated group and reached 66.97 ± 4.27% in the 0.5 μg vaccine-treated group. These data demonstrate that the 38-mRNA nanoparticle vaccine significantly enhances the proliferative capacity of CD38-targeted CAR-T cells in a dose-dependent manner (Figure 4H-I).

Based on established classification criteria for memory T cell subsets in the literature 9, we further analyzed the effect of the 38-mRNA vaccine on the memory differentiation status of Ko-38CAR cells. Within the CD4⁺ CAR-T cell subset, compared with the Ko-38CAR control group (mean ± SD 10.35 ± 3.25%), the vaccine-treated groups exhibited a dose-dependent and statistically significant increase in the proportion of central memory T cells (CD45RA⁻CCR7⁺) (mean: 0μg, 13.65 ± 2.58%; 0.1 μg, 17.38 ± 1.53%; 0.5 μg, 21.93 ± 2.29%). The proportion of naïve-like T cells (CD45RA⁺CCR7⁺) showed an upward trend but did not reach statistical significance, whereas the proportion of terminally differentiated effector T cells (CD45RA⁺CCR7⁻) was significantly reduced (mean: Ko-38CAR, 45.08 ± 5.07%; 0 μg, 42.03 ± 6.04%; 0.1 μg, 36.18 ± 6.58%; 0.5 μg, 31.78 ± 3.34%), with statistical significance (Figure 4J-K). In the CD8⁺ CAR-T cell subset, no significant differences were observed in the proportion of central memory T cells among the groups. The naïve-like T cell proportion exhibited a statistically significant increase (mean ± SD: Ko-38 CAR, 16.55 ± 7.01%; 0 μg, 22.53 ± 10.92%; 0.1 μg, 32.80 ± 8.82%; 0.5 μg, 37.83 ± 7.21%), consistent with the trend observed in the CD4⁺ subset. The proportion of terminally differentiated effector T cells also showed a downward trend but did not reach the threshold of statistical significance (Figure 4L). Collectively, these results indicate that the 38-mRNAvaccine induces diversified remodeling of memory phenotypes in CD38-targeted CAR-T cells.

To further investigate the impact of the 38-mRNA vaccine on the activation status and exhaustion phenotype of CD38-targeted CAR-T cells, Ko-38CAR cells were co-cultured for 24 hours with dendritic cells pretreated with varying doses of the vaccine. Flow cytometry was then employed to assess the expression levels of the activation marker CD69 and the immune checkpoint molecule PD-1. The results demonstrated that the 38-mRNA vaccine promoted CAR-T cell activation in a dose-dependent manner: in both CD4⁺ and CD8⁺ CAR-T cell subsets, CD69 expression was significantly upregulated with increasing vaccine doses (Figure 5A-B). Concurrently, PD-1 expression also exhibited a dose-dependent increasing trend, which may be associated with CAR-T cell activation (Figure 5C-D).

To examine the impact of the 38-mRNA vaccine on the cytokine secretion of CD38 CAR-T cells, we employed cytometric bead array (CBA) technology to quantitatively analyze the cytokines in the culture supernatants of each group of CAR-T cells after 24 h of co-culture. The results revealed an upregulation in the secretion of multiple effector-associated cytokines, including IFN-γ, IL-4, TNF-α, IL-2, IL-8, and IL-10. Notably, compared with the Ko-38CAR alone culture group (mean ± SD 12.45 ± 1.61 pg/mL), IL-2 secretion was markedly elevated in the 0.5 μg vaccine-treated group (mean 2349.71 ± 169.90 pg/mL), representing an approximately 195-fold increase; an approximately 2.5-fold increase was observed in the Ko-38CAR+DC (0 μg) group (mean 31.57 ± 2.77 pg/mL), and an approximately 33-fold increase was observed in the 0.1 μg vaccine-treated group (mean 421.40 ± 20.87 pg/mL). In addition, a significant decrease in the secretion of IL-6 and IL-1β was observed after co-culture of CAR-T cells with dendritic cells loaded with the 38-mRNA vaccine (Figure 5E).

To further evaluate the impact of the 38-mRNA vaccine on the tumor cell killing capacity of CD38 CAR-T cells, the *in vitro* cytotoxicity assay was designed and conducted (Figure 6A–C). We observed that the 38-mRNA vaccine enhanced the killing efficacy of CD38-targeted CAR-T cells against both MOLM-13 and THP-1 cell lines in a dose-dependent manner. Notably, under high effector-to-target ratio (1:5) conditions, the high-dose vaccine combination group exhibited more potent tumor cell clearance capacity (MOLM-13 mean ± SD: Ko-38CAR, 20.43 ± 3.39%; 0 μg, 24.03 ± 3.47%; 0.1 μg, 44.38 ± 5.93%; 0.5 μg, 54.25 ± 3.49%; THP-1 mean ± SD: Ko-38CAR, 13.33 ± 1.54%; 0 μg, 43.00 ± 1.50%; 0.1 μg, 46.25 ± 3.52%; 0.5 μg, 86.37 ± 3.54%) (Figure 6D–G). To determine the impact of the vaccine on cytokine release during the enhancement of CD38 CAR-T cell antitumor efficacy, quantitative cytokine analysis was performed. The results revealed that IL-2 and IFN-γ secretion remained at high levels under high-dose vaccine treatment and high effector-to-target ratio conditions (Figure S2A, C), whereas TNF-α secretion remained generally stable but declined slightly under high effector-to-target ratios (Figure S2D). Notably, IL-4 secretion was maintained at the highest level in the high-dose vaccine combination group under high effector-to-target ratio conditions (Figure S2B).

To further investigate the impact of the 38-mRNA vaccine on the killing of primary AML cells by CD38 CAR-T cells, primary blasts derived from two patients with high-risk AML were co-cultured with CD38 CAR-T cells, according to the method described 14. The results demonstrated that the 38-mRNA vaccine dose-dependently enhanced the cytotoxic effect of CD38 CAR-T cells against those primary CD38⁺ AML blasts (Figure S3A–B).

### The 38-mRNA nanoparticle vaccine alters the transcriptomic profile and chromatin accessibility landscape of CD38-targeted CAR-T cells

To systematically elucidate the underlying molecular mechanisms by which the 38-mRNA nanoparticle vaccine enhances CD38 CAR-T cell function, we co-cultured Ko-38CAR cells with dendritic cells pretreated with or without 0.5 μg of the 38-mRNA vaccine for 24 hours. CAR-positive T cells were subsequently sorted and divided into three groups based on co-culture conditions: Ko-38CAR alone (T), Ko-38CAR co-cultured with unloaded dendritic cells (T0), and Ko-38CAR co-cultured with 0.5 μg vaccine-loaded dendritic cells (T05). Transcriptome sequencing analysis was then performed. Principal component analysis and differential gene clustering revealed distinct separation of the global transcriptional profiles among the three groups (Figure 7A-C). Trend analysis of all differentially expressed genes identified six major expression patterns. Based on the dose-dependent functional enhancement observed in the preceding experiments, we focused on gene clusters exhibiting sustained upregulation along the T, T0, T05 sequence, namely cluster 3 and cluster 4 (Figure 7D).

GO and KEGG pathway enrichment analysis of cluster 3 revealed that this gene cluster is predominantly involved in biological processes such as the *JAK-STAT* signaling pathway, cytokine response, and ATP energy metabolism (Figure 7E-G). Heatmap analysis visually demonstrated that genes associated with the *JAK-STAT* signaling axis (e.g., *OSM*, *LIF, BCL2*, *CSF2*) were markedly upregulated in the T05 group (Figure 7H). Among these, overexpression of the anti-apoptotic gene *BCL2* has been previously reported to be closely associated with enhanced *in vivo* survival and persistence of CAR-T cells 15, 16. Consistently, cluster 3 was also enriched for genes encoding activation markers (e.g., *ICOS*), various effector cytokines (e.g., *IFNG*, *TNF*, *IL2*), and cytotoxic effector molecules (e.g., *GZMH*, *GZMB*) (Figure 7I-K). Notably, the upregulation of* IL2*,* IFNG*, and *TNF* observed at the transcriptomic levels was highly consistent with the enhanced cytokine secretion detected at the protein level by CBA in preceding experiments, thereby corroborating the potentiating effect of the vaccine on CAR-T cell effector function at the transcriptional level.

Functional annotation revealed that Cluster 4 was significantly enriched in pathways related to cell cycle regulation, DNA replication, DNA repair, and arginine and proline metabolism (Figure 7L–N). Heatmap analysis further confirmed that key genes in the arginine and proline metabolism pathway and the polyamine metabolism pathway (such as *ODC1*) were markedly upregulated (Figure 7O); in addition, multiple core transcription factors driving cell cycle progression and proliferation (e.g., *E2F1*,* CDC7*, *PCNA*) showed significantly elevated expression in the T05 group (Figure 7P). These transcriptomic features provide a direct mechanistic explanation, from the perspectives of molecular regulation and metabolism, for the previously observed dose-dependent enhancement of CAR-T cell proliferative capacity by the 38-mRNA vaccine.

We further employed ATAC-seq technology to systematically evaluate the impact of the 38-mRNA nanoparticle vaccine on the chromatin accessibility landscape of CD38-targeted CAR-T cells. Quality control data demonstrated that ATAC signals in all samples exhibited a characteristic unimodal enrichment pattern near transcription start sites, confirming effective capture of open chromatin regions. The signal distribution patterns among the different treatment groups (T, T0, T05) and their biological replicates were highly consistent, ensuring the reliability and reproducibility of subsequent analyses (Figure S4A). Building on this, we further observed a gradual increase in ATAC signal enrichment within promoter regions across the T, T0, and T05 groups, suggesting that vaccine treatment may broadly enhance chromatin accessibility at the promoter regions of relevant genes (Figure S4B-C).

To enable systematic cross-omics comparison with the transcriptomic analysis, we applied the same trend analysis strategy to cluster all differentially accessible regions. Based on the functional phenotypic enhancement observed previously, we focused on the gene cluster (Cluster 3) exhibiting a sustained increase in chromatin accessibility along the T, T0, T05 sequence (Figure S5A-B, Figure S6). GO functional enrichment analysis revealed that the open chromatin regions within this cluster were predominantly associated with key biological processes, including regulation of cell activation, positive regulation of the *JNK* signaling pathway, cell cycle progression, and the *JAK-STAT* signaling cascade (Figure S7). KEGG pathway enrichment analysis further demonstrated that chromatin accessibility at loci related to the *MAPK* signaling pathway, *TNF* signaling pathway, and *JAK-STAT* signaling pathway was significantly enhanced following vaccine treatment (Figure S8).

Notably, heatmap visualization analysis revealed that multiple gene loci closely associated with CAR-T cell effector function—including activation marker genes (*CD69*, *ICOS*) and genes encoding cytotoxic effector molecules (*NKG7*, *IFNG*, *GZMH*, *GZMB*)—exhibited markedly increased chromatin accessibility in the T05 group (Figures S9-10). These epigenomic findings are highly concordant with the upregulation of corresponding gene expression observed in the preceding transcriptomic analysis and the enhanced secretion of effector molecules detected by CBA, indicating that the 38-mRNA vaccine achieves comprehensive and durable enhancement of CAR-T cell function by epigenetically modulating their effector differentiation potential.

### The 38-mRNA vaccine promotes *in vivo* proliferation of CD38-targeted CAR-T cells and antitumor efficacy in an AML xenograft model

For *in vivo* efficacy evaluation, based on the predominant expression of the 38-mRNA vaccine in antigen-presenting cells, an immunodeficient mouse strain (NOD.Cg-*Prkdc*^scid^*Il2rg*^em1Smoc^) characterized by combined T cell, B cell, and NK cell deficiency while retaining functional macrophages and dendritic cells was utilized to meet the experimental requirement for functional antigen-presenting cells. With reference to the previously reported experimental protocols 8, three CAR-T + vaccine dose groups (empty vector control (Ctrl), 10 μg, and 20 μg) were established to assess the capacity of the vaccine to enhance the *in vivo* proliferative potential of CD38-targeted CAR-T cells and the safety of the combination therapy strategy (Figure S11A).

Flow cytometry analysis was performed to detect CD3 and CD45 double-positive cells, which were used as surrogate markers for tracking CAR-T cells to analyze their proportional distribution across various tissues 17. The results showed that CAR-T cells were most abundant in the SP and least enriched in the BM. Notably, the proportions of CAR-T cells in both the SP and BM increased in a dose-dependent manner with escalating doses of the 38-mRNA nanoparticle vaccine, with the highest proportions observed in the 20 μg dose group (SP 2.55 ± 1.17%, PB 4.47 ± 0.60%, BM 1.09 ± 0.16%) (Figure S11C-F).

Regarding safety, we focused on the potential CAR-T toxicity that may arise from off-target delivery of the vaccine to the liver. Serum biochemical analysis revealed that AST and ALT levels were transiently elevated on day 8 and returned to normal by day 14 (Figure S11H). More importantly, TUNEL staining of liver tissues collected on day 14 showed no evident hepatocellular apoptosis. Furthermore, no significant decline in body weight was observed in any group throughout the experimental period; instead, an overall slight weight gain was noted, indicating that the combination regimen was generally well tolerated (Figure S11B, G).

Based on the preceding observation that the 38-mRNA nanoparticle vaccine promotes CAR-T cell expansion *in vivo* in a dose-dependent manner, we further evaluated the impact of this combination strategy on the *in vivo* antitumor efficacy. An AML cell line-derived xenograft model was established by tail vein injection of luciferase-labeled THP-1 cells into NSG mice, and tumor burden was dynamically monitored using *in vivo* bioluminescence imaging (Figure 8A).

*In vivo* imaging results showed that during the early treatment phase (approximately day 12), both the Ko-38CAR group and the Ko-38CAR+20 μg Vaccine group effectively suppressed tumor growth, albeit with no significant difference between them. However, as the observation time proceeded, the Ko-38CAR+20 μg Vaccine group exhibited more sustained and superior antitumor efficacy, and the tumor burden in this group remained persistently low, whereas the tumor-suppressive effect in the Ko-38CAR group gradually diminished over time (Figure 8B). Survival analysis further confirmed that combination therapy apparently prolonged the median survival of tumor-bearing mice, with a statistically significant difference (Figure 8D-E). Throughout the observation period, no significant decline in body weight was observed in any group, further supporting the favorable tolerability of the combination regimen (Figure 8C).

Histopathological evaluation provided direct evidence supporting the above functional observations. At the experimental endpoint, liver tissues were subjected to H&E staining and CD38 immunohistochemical staining. The results showed extensive infiltration of CD38-positive tumor cells in the livers of mice in the NT group, limited infiltration in the Ko-38CAR group, and, in striking contrast, virtually undetectable CD38-positive tumor cells in the livers of mice in the Ko-38CAR + 20 μg vaccine group (Figure 8F-G), corroborating the profound tumor clearance achieved by the combination therapy at the histological level.

Complete blood count analysis was performed on peripheral blood. The results showed that absolute neutrophil and lymphocyte counts in the peripheral blood of the Ko-38CAR+20 μg Vaccine group exhibited statistically significant increases (Figure 8H).

## Discussion

Relapse and suboptimal therapeutic efficacy remain major clinical challenges for the application of chimeric antigen receptor T-cell therapy in patients with R/R AML 3, 18, 19. In stark contrast to the breakthrough achieved in multiple myeloma and B-cell lymphoblastic leukemia, the therapeutic benefit of CAR-T cell therapy in R/R AML has consistently fallen short of expectations 20, 21. Although our group's prior studies have preliminarily demonstrated the acceptable safety profile and certain therapeutic activity of CD38-targeted CAR-T cell strategies in R/R AML, disease recurrence remains a prominent issue. Notably, AML blast cells from relapsed patients continue to exhibit CD38 antigen expression 4. This clinical reality underscores the urgent need to further augment the effector function and long-term persistence of CD38 CAR-T cells.

The experimental data consistently demonstrate that this vaccine significantly augments the activation level, proliferative potential, effector function, and *in vivo* persistence of CD38 CAR-T cells. These findings confirm that a dual-activation strategy—combining exogenous antigen presentation in its native conformation with costimulatory signals derived from antigen-presenting cells—can effectively potentiate the antitumor efficacy of CAR-T cells in R/R AML. Collectively, the above findings provide a new strategic reference and mechanistic basis for improving the clinical efficacy of CAR-T cell therapy in R/R AML The results indicate that the 38-mRNA vaccine, by virtue of the inherent phagocytic tropism of the lipid nanoparticle carrier for antigen-presenting cells, can efficiently target the splenic APC population. This targeting feature is critically important for maximizing the probability of contact between CAR-T cells and the vaccine. Existing literature has established that adoptively transferred CAR-T cells exhibit a biological propensity for preferential homing to secondary lymphoid organs, particularly the spleen, *in vivo* 22.

The antigen expression mediated by the 38-mRNA nanoparticle vaccine exhibits a characteristic pulsatile kinetic profile: CD38 expression peaks at 24 hours post-administration and declines markedly by 48 hours. On the one hand, transient antigenic stimulation is sufficient to effectively initiate CAR-T cell activation and expansion programs while circumventing the excessive T-cell activation and subsequent functional exhaustion that may be induced by persistent antigen exposure. On the other hand, the rapid decline in antigen expression substantially mitigates the potential off-target risks arising from vaccine misdistribution or ectopic expression.

In this study, CD38-targeted CAR-T cells were constructed using the single-chain variable fragment derived from daratumumab. Phenotypic comparison between CD38-knockout (Ko-38CAR) and non-knockout (38CAR) CAR-T cells revealed that by day 7 of culture, 38CAR cells exhibited nearly 100% CAR positivity but near-zero CD38 expression, resembling the fratricide-driven selection reported for CD7 CAR-T cells 23, 24. This process impairs cell yield and functional fitness, thereby compromising subsequent expansion and effector capacity. Thus, CD38 knockout or blockade is necessary to restore the function of this CAR-T cell type.

According to previous reports, declining tumor burden in later stages of CAR-T cell therapy may lead to insufficient antigen stimulation, resulting in restricted CAR-T cell expansion and numerical decline, thereby weakening immune surveillance, compromising remission depth, and associating with disease relapse 6, 25, 26. Thus, maintaining an adequate CAR-T cell number is critical for therapeutic efficacy. This study demonstrates that the 38-mRNA vaccine promotes CD38 CAR-T cell expansion both *in vitro* and *in vivo*, an effect that occurs independently of tumor cell presence. This finding suggests that the vaccine may support CAR-T cell numerical maintenance via an exogenous antigen pathway, thereby restoring immune surveillance and enabling deeper therapeutic remission in R/R AML patients.

The induction of memory CAR-T cells has long remained a challenge in the field of CAR-T cell therapy. This study demonstrates that the 38-mRNA nanoparticle vaccine can promote the differentiation of CAR-T cells into memory subsets with diverse phenotypes. This capacity may involve a dual mechanism: first, activation of the intracellular 4-1BB-CD3ζ signaling axis of the CAR molecule via CD38 antigen presentation; second, facilitation of physical contact and signal crosstalk between dendritic cells and CAR-T cells, thereby providing additional costimulatory signals 8, 9.

Regarding cytokine secretion, following co-culture of CAR-T cells with DCs transfected with different vaccine doses, markedly increased IL-2 secretion was observed alongside significantly elevated levels of effector cytokines (IFN-γ, TNF-α). Given that IL-2 is a key regulator of CAR-T cell expansion, this finding may provide a partial mechanistic explanation for the capacity of the vaccine to promote CAR-T cell proliferation 16, 27. Previous studies have demonstrated that elevated IL-2 and IFN-γ levels are positively correlated with favorable prognosis in patients with hematologic malignancies treated with CAR-T cell therapy 27. Furthermore, increased IL-4 secretion was also noted. According to other reports, elevated IL-4 levels are associated with the maintenance of long-term responses in clinical CAR-T cell therapy, and the addition of IL-4 during antigen-specific activation can mitigate CAR-T cell dysfunction and enhance their fitness at both the transcriptomic and epigenomic levels 28, 29. Finally, we observed that, compared with unloaded dendritic cells, dendritic cells loaded with the vaccine exhibited decreased secretion of IL-6 and IL-1β in the supernatant after co-culture with CAR-T cells. Given that IL-1β and IL-6 are closely associated with the development of cytokine release syndrome (CRS), this finding may be beneficial for improving the safety of vaccine-combined CAR-T therapy 30-32.

When evaluating the impact of the 38-mRNA nanoparticle vaccine on the antileukemic efficacy of CD38 CAR-T cells, the experimental protocol was adjusted to account for proliferation as a potential variable. Specifically, the co-culture duration of vaccine-pretreated dendritic cells with CAR-T cells was extended to 96 hours—consistent with the timeframe of the proliferation assay—prior to the addition of target cells for cytotoxicity assessment. Under these conditions, the antileukemic efficacy of CAR-T cells increased with escalating vaccine doses. These findings suggest that enhancement of CAR-T cell expansion may play an important role in this antigen-boosting strategy.

The 38-mRNA vaccine profoundly reshaped the transcriptional profile and chromatin accessibility landscape of CD38 CAR-T cells and promoted the enrichment of both *JAK-STAT* and *MAPK* signaling pathways. Activation of the *JAK-STAT* pathway, a core conduit for cytokines such as IL-2, was closely associated with enhanced IL-2 secretion, T cell survival, and proliferation 16, 33. Activation of the *MAPK* pathway, potentially triggered via the 4-1BB costimulatory domain of the CAR molecule, contributed to the regulation of T cell activation and effector function 34, 35. Furthermore, the vaccine upregulated the anti-apoptotic gene *BCL2,* thereby enhancing CAR-T cell survival and *in vivo* persistence 15.

Concurrently, the expression of the costimulatory molecule ICOS and granzyme-related genes (*GZMB, GZMH*) in CAR-T cells was also augmented by the vaccine. Multi-omics analyses further suggested that the 38-mRNA vaccine may promote CAR-T cell proliferation through the coordinated upregulation of multiple pathways involved in DNA replication, DNA repair, and cell cycle progression. Within the cell cycle pathway, the expression of several genes that positively regulate proliferation (e.g., *CDK4, CDK6*) was significantly elevated; these genes are involved in enhancing DNA replication licensing, driving cell cycle progression (including G1/S and G2/M transitions), and maintaining mitotic fidelity. In addition, genes associated with the polyamine metabolism pathway, such as *ODC1*, were markedly upregulated. Previous studies have demonstrated that polyamine metabolism plays a critical role in T cell activation and clonal expansion by supporting nucleic acid and protein synthesis, stabilizing chromatin structure, and regulating translation 36. Moreover, polyamines help maintain the metabolic fitness of T cells by modulating mitochondrial function and oxidative phosphorylation. These findings provide further mechanistic insights into how the vaccine potentiates CAR-T cell function from a metabolic perspective 37.

The results demonstrated that the *in vivo* antileukemic efficacy of CAR-T cells was effectively enhanced by the 38-mRNA nanoparticle vaccine, and further evaluation in additional CDX models and humanized models will be conducted in subsequent studies. Although the combination therapy was accompanied by transient elevations in liver function parameters (AST and ALT), TUNEL staining of liver tissues revealed no evident increase in hepatocellular apoptosis, and neither mouse body weight nor survival time was significantly affected. Further improvements in the organ-targeting specificity of the delivery system may be achieved through subsequent optimization of the lipid nanoparticle formulation and the route of administration. This combination therapeutic strategy will continue to be refined by our group in future work.

This study focused on the dendritic cell-mediated potentiating effects of the 38-mRNA nanoparticle vaccine. Since CD38 antigen presentation also occurred in murine macrophages following the vaccine injection, we speculated that CD38 antigen-loaded macrophages may also play a role in boosting the CAR. Indeed, our preliminarily results showed that macrophages could significantly enhance the proliferative capacity of CD38 CAR-T cells (Figure S12A–B) and promote their activation (Figure S12C–D), supporting that macrophages may possess a CAR-T potentiating capacity similar to that of dendritic cells. However, a more comprehensive investigation is still warranted to firmly validate the involvement of macrophages, which will be conducted in an independent study in the future.

## Conclusion

In summary, the 38-mRNA nanoparticle vaccine developed in this study enables pulsatile delivery of CD38 antigen via splenic antigen-presenting cells and, through dual activation of CAR and costimulatory signaling, significantly enhances the proliferative capacity, effector function, and *in vivo* persistence of CD38 CAR-T cells. This strategy offers a novel interventional approach to mitigate CAR-T cell functional decline and prevent disease relapse in R/R AML.

## Supplementary Material

Supplementary methods and figures.

Supplementary ATAC-SEQ data sheet.

Supplementary RNA-SEQ cluster 3 genes data sheet.

Supplementary RNA-SEQ cluster 4 genes data sheet.

## Figures and Tables

**Figure 1:**
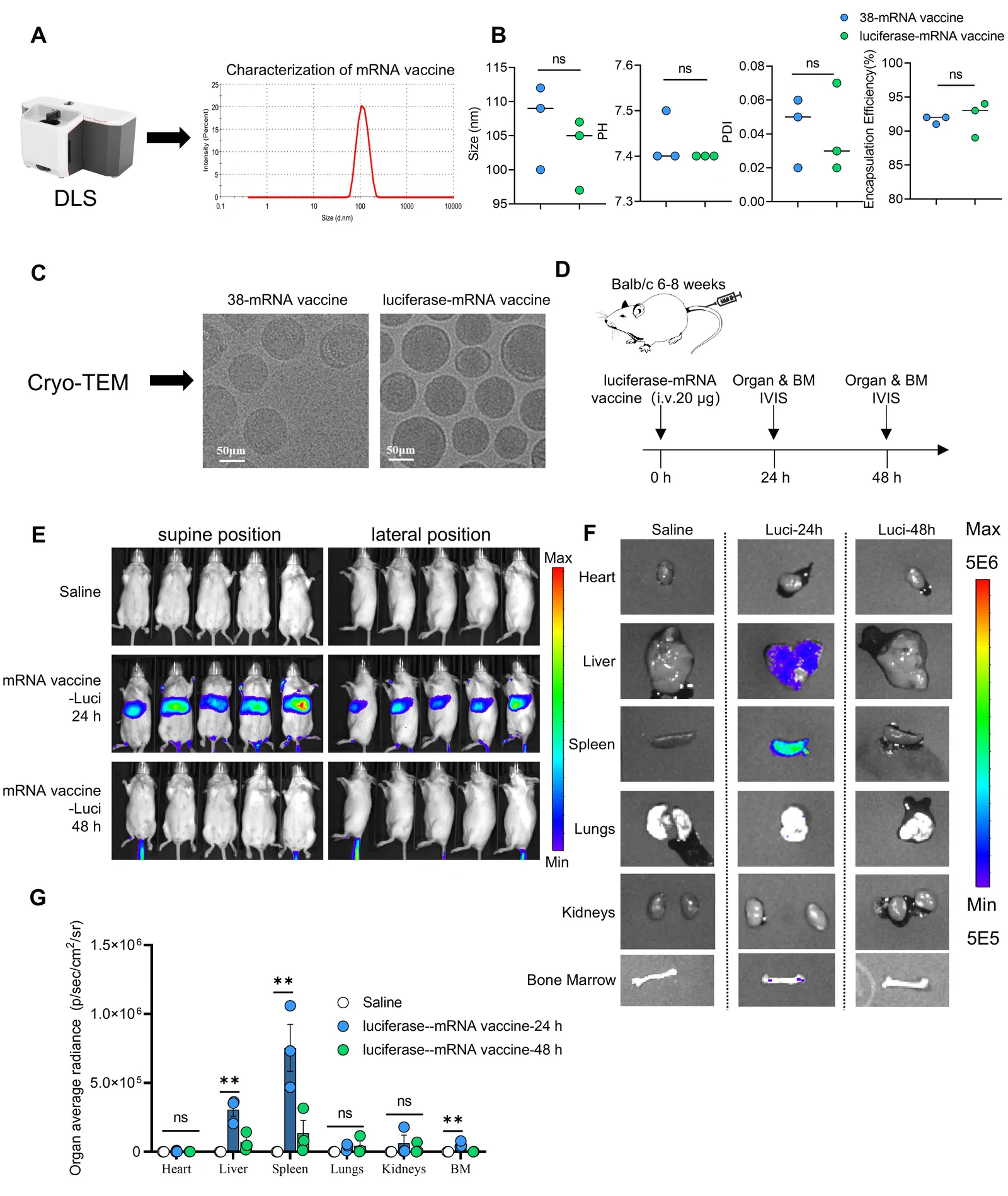
** Preparation, characterization, and *in vivo* tracking of the 38-mRNA vaccine.** (A) Schematic of DLS detection of ionizable LNPs encapsulating CD38 mRNA or luciferase mRNA. (B) Particle size, pH, PDI, and encapsulation efficiency of the two mRNA LNP vaccines measured by DLS (N = 3). (C) Representative cryo-EM images of CD38 mRNA and luciferase mRNA LNP vaccines (scale bar = 50 nm). (D) Schematic of the experimental protocol: BALB/c mice (6–8 weeks old) were injected i.v. with luciferase mRNA LNP vaccine or an equivalent volume of saline (20 μg/mouse) at 0 h. *In vivo* bioluminescence imaging and organ collection were performed at 24 h and 48 h post-injection. (E) Representative *in vivo* bioluminescence images of the saline group and mRNA LNP vaccine distribution at 24 h and 48 h post-injection. (F–G) *Ex vivo* bioluminescence images of organ and bone marrow distribution at 24 h and quantitative analysis of fluorescence signals in major organs (N = 3). All data are presented as mean ± SD. ns, not significant; **, *p* < 0.01.

**Figure 2:**
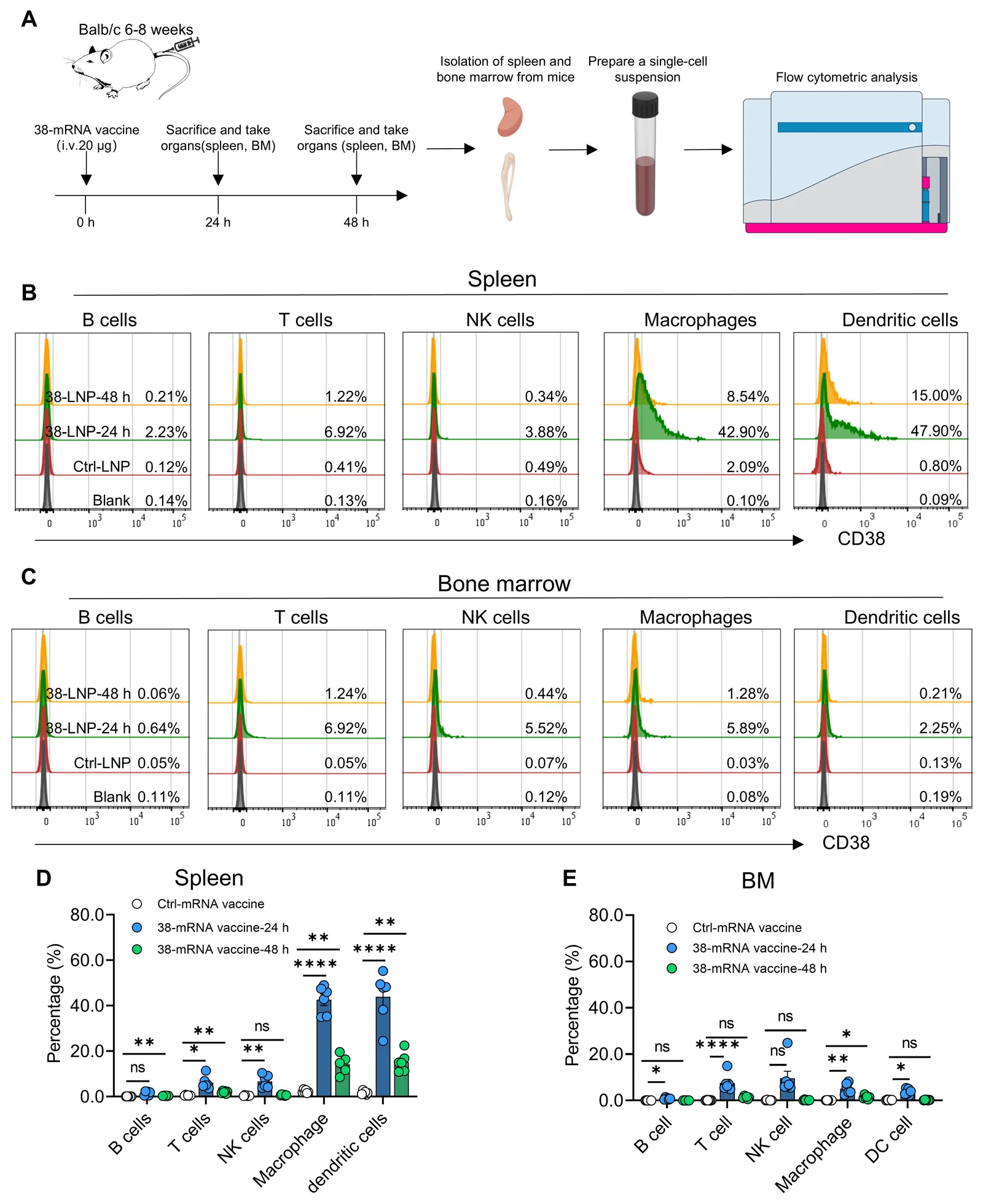
***In vivo* organ and cellular distribution of the 38-mRNA vaccine.** (A) Schematic of the experimental protocol: BALB/c mice (6–8 weeks old) were injected i.v. with the 38-mRNA vaccine (20 μg/mouse) via the tail vein. Mice were sacrificed at 24 h and 48 h post-injection, and the spleen and bone marrow were collected to prepare single-cell suspensions. CD38 expression in various immune cell subsets was detected by flow cytometry. (B, D) Representative flow cytometry plots and quantitative analysis of CD38 expression in B cells, T cells, NK cells, macrophages, and dendritic cells in the spleen (N = 6). (C, E) Representative flow cytometry plots and quantitative analysis of CD38 expression in the above immune cell subsets in the bone marrow (N = 6). All data are presented as mean ± SD. ns, not significant; *, *p* < 0.05; **, *p* < 0.01; ****, *p* = 0.0001.

**Figure 3:**
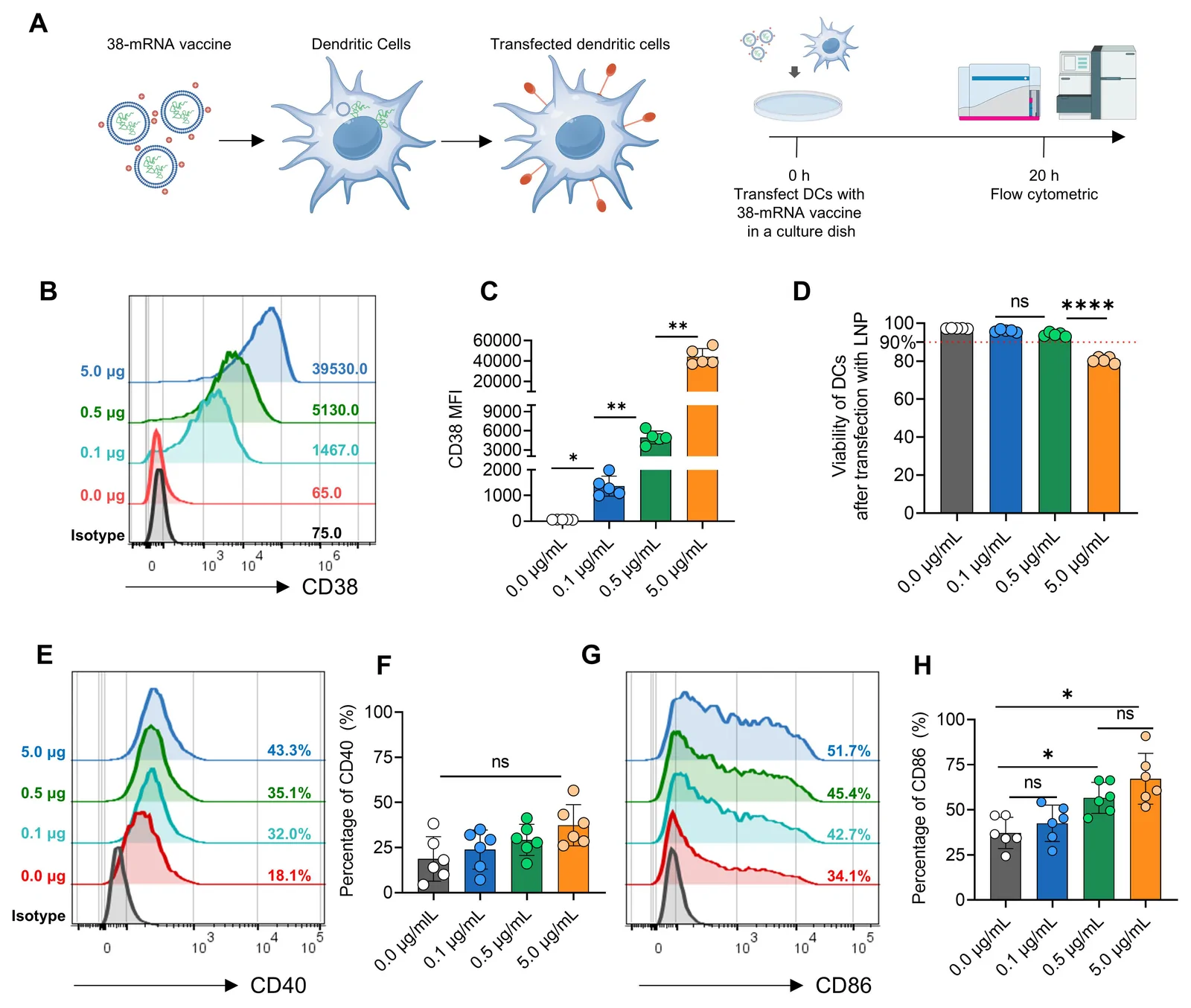
***In vitro* transfection efficiency and safety assessment of the 38-mRNA vaccine in dendritic cells.** (A) Schematic of the experimental protocol: Different doses of the 38-mRNA vaccine were added to dendritic cell cultures and transfected for 20 h, followed by flow cytometric detection of CD38 expression, cell viability, and costimulatory molecule expression. (B-C) Representative flow cytometry plots and quantitative analysis of CD38 expression in dendritic cells treated with different vaccine doses (N = 5). (D) Quantitative analysis of dendritic cell viability in different dose groups (N = 5). (E-H) Expression rates of the costimulatory molecules CD40 and CD86 in dendritic cells treated with different vaccine doses (N = 6). All data are presented as mean ± SD. ns, not significant; *, *p* < 0.05; **, *p* < 0.01; ****,* p* = 0.0001.

**Figure 4:**
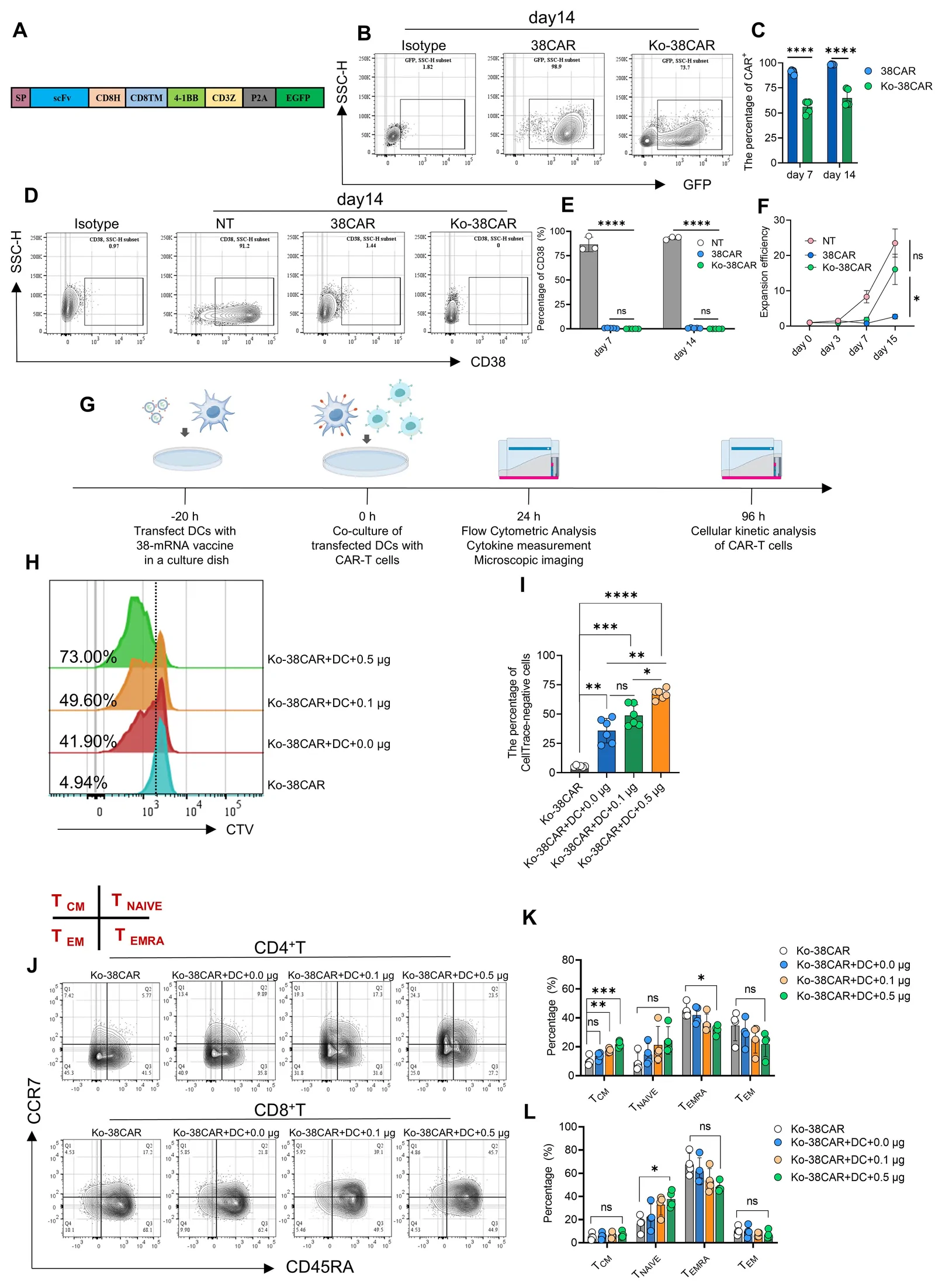
***In vitro* analysis of the effects of the 38-mRNA vaccine on CD38 CAR-T cell proliferation and memory phenotype.** (A) Schematic of the CD38-targeted chimeric antigen receptor construct. (B-C) Representative flow cytometry plots and quantitative analysis of CAR transfection efficiency in CD38-knockout (Ko-38CAR) and non-knockout (38CAR) CAR-T cells on days 7 and 14 (N = 5). (D-E) Representative flow cytometry plots and quantitative analysis of CD38 expression in Ko-38CAR and 38CAR cells on days 7 and 14. (F) Proliferation curves of non-transduced control (NT), 38CAR, and Ko-38CAR cells. (G) Schematic of the experimental protocol: Dendritic cells were transfected with different doses of the 38-mRNA vaccine at -20 h, and transfection was terminated at 0 h. Dendritic cells were then co-cultured with T cells at a 1:10 ratio. CAR-T cell phenotype was assessed by flow cytometry after 24 h of co-culture, and CAR-T cell proliferation was measured after 96 h. (H-I) Representative flow cytometry plots and quantitative analysis of the proliferation rate of CTV-labeled CD38 CAR-T cells (N = 6). (J-K) Flow cytometric analysis of memory phenotypes in CD4⁺ and CD8⁺ CD38 CAR-T cells after 24 h of co-culture (T_CM,_ CCR7⁺CD45RA⁻; T_NAIVE_, CCR7⁺CD45RA⁺; T_EM_, CCR7⁻CD45RA⁻; T_EMRA_, CCR7⁻CD45RA⁺) (N=4). All data are presented as mean ± SD. ns, not significant; *, *p* < 0.05; **, *p* < 0.01; ***, *p* = 0.001; ****, *p* = 0.0001.

**Figure 5:**
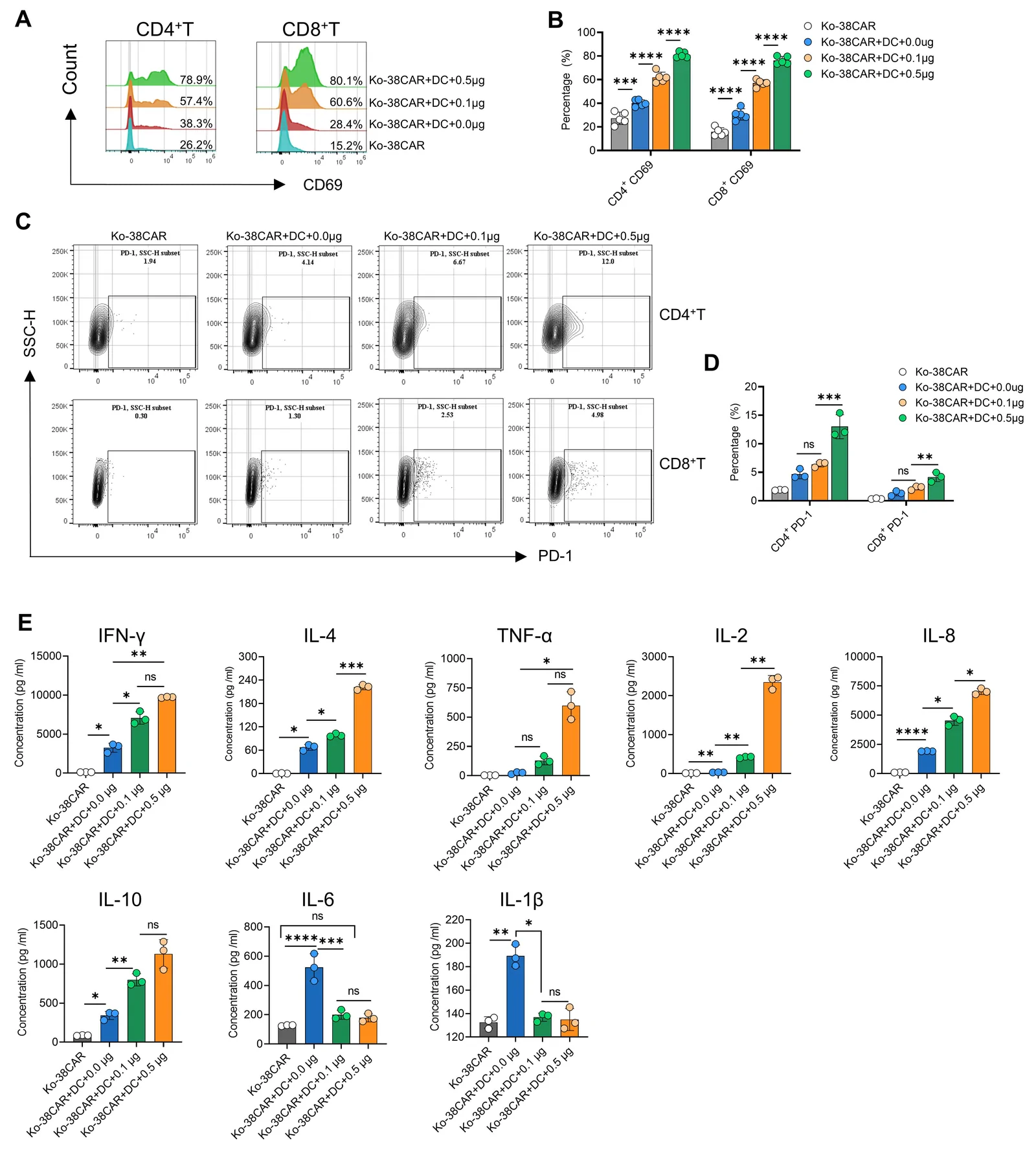
** Effects of the 38-mRNA vaccine on CD38 CAR-T cell activation, exhaustion, and cytokine release.** (A-B) Flow cytometric analysis of the effect of the 38-mRNA vaccine on the expression of the activation marker CD69 in CD4⁺ and CD8⁺ CD38 CAR-T cells (N = 5). (C-D) Flow cytometric analysis of the effect of the 38-mRNA vaccine on the expression of the exhaustion marker PD-1 in CD4⁺ and CD8⁺ CD38 CAR-T cells (N = 3). (E) Cytometric bead array analysis of cytokine levels (IFN-γ, IL-4, TNF-α, IL-2, IL-8, IL-10, IL-6, IL-1β) in culture supernatants after 24 h of co-culture (N = 3). All data are presented as mean ± SD. ns, not significant; *, *p* < 0.05; **, *p* < 0.01; ***, *p* = 0.001; ****, *p* = 0.0001.

**Figure 6:**
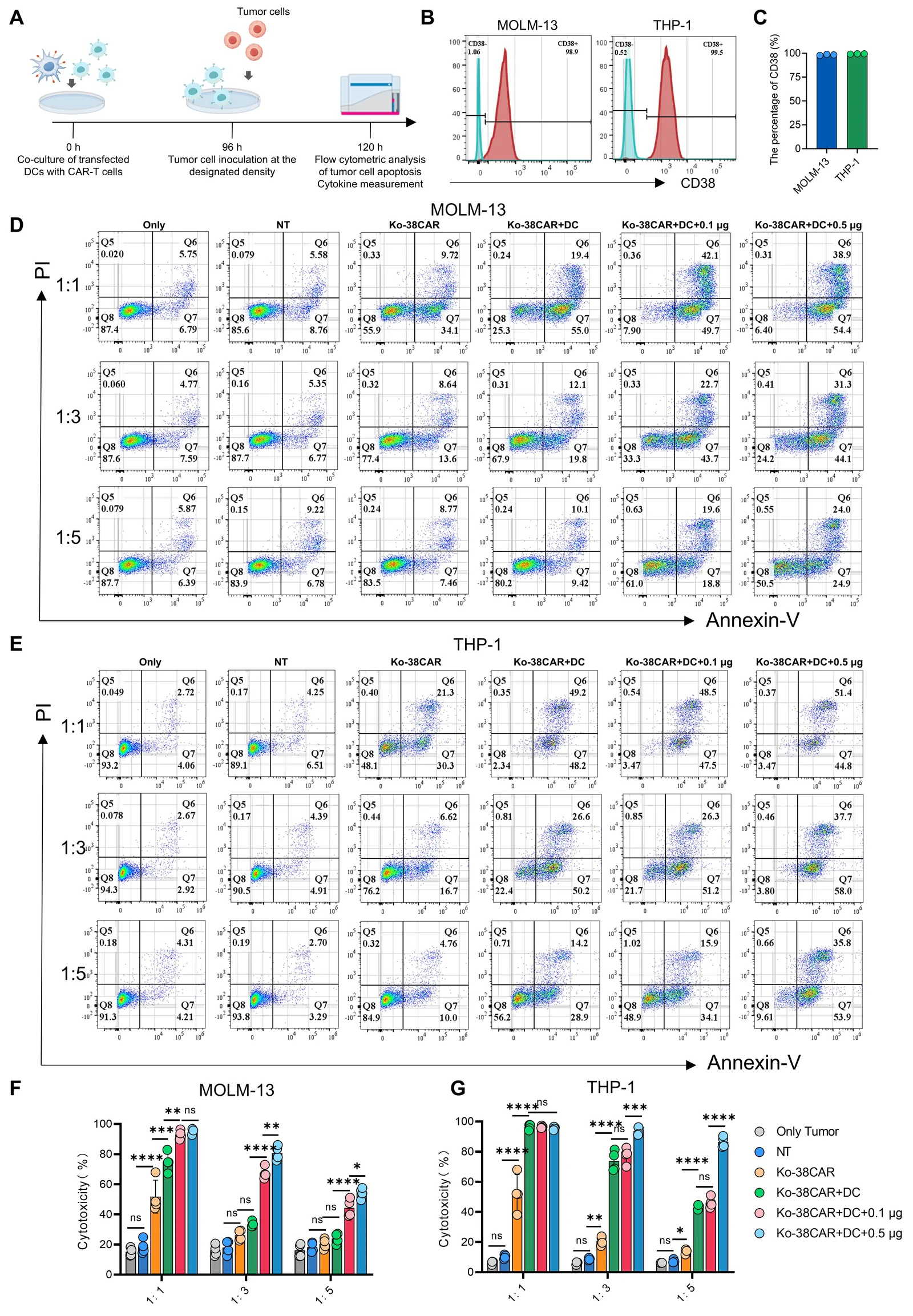
The 38-mRNA vaccine enhances the killing efficacy of CD38 CAR-T cells against CD38^+^ target cell lines. (A) Schematic of the experimental protocol: Dendritic cells pretreated with different doses of the 38-mRNA vaccine were co-cultured with CD38 CAR-T cells. Target cells were then added at different effector-to-target ratios and incubated for an additional 16–18 h. Target cell apoptosis was detected by flow cytometry. (B-C) Representative histograms of CD38 expression on the AML cell lines THP-1 and MOLM-13 detected by flow cytometry (N = 3). (D-G) Assessment of the potentiating effect of different doses of the 38-mRNA vaccine on the killing capacity of CD38 CAR-T cells based on flow cytometric detection of target cell apoptosis (N = 4). All data are presented as mean ± SD. ns, not significant; *, *p* < 0.05; **, *p* < 0.01; ***, *p* = 0.001; ****, *p* = 0.0001.

**Figure 7:**
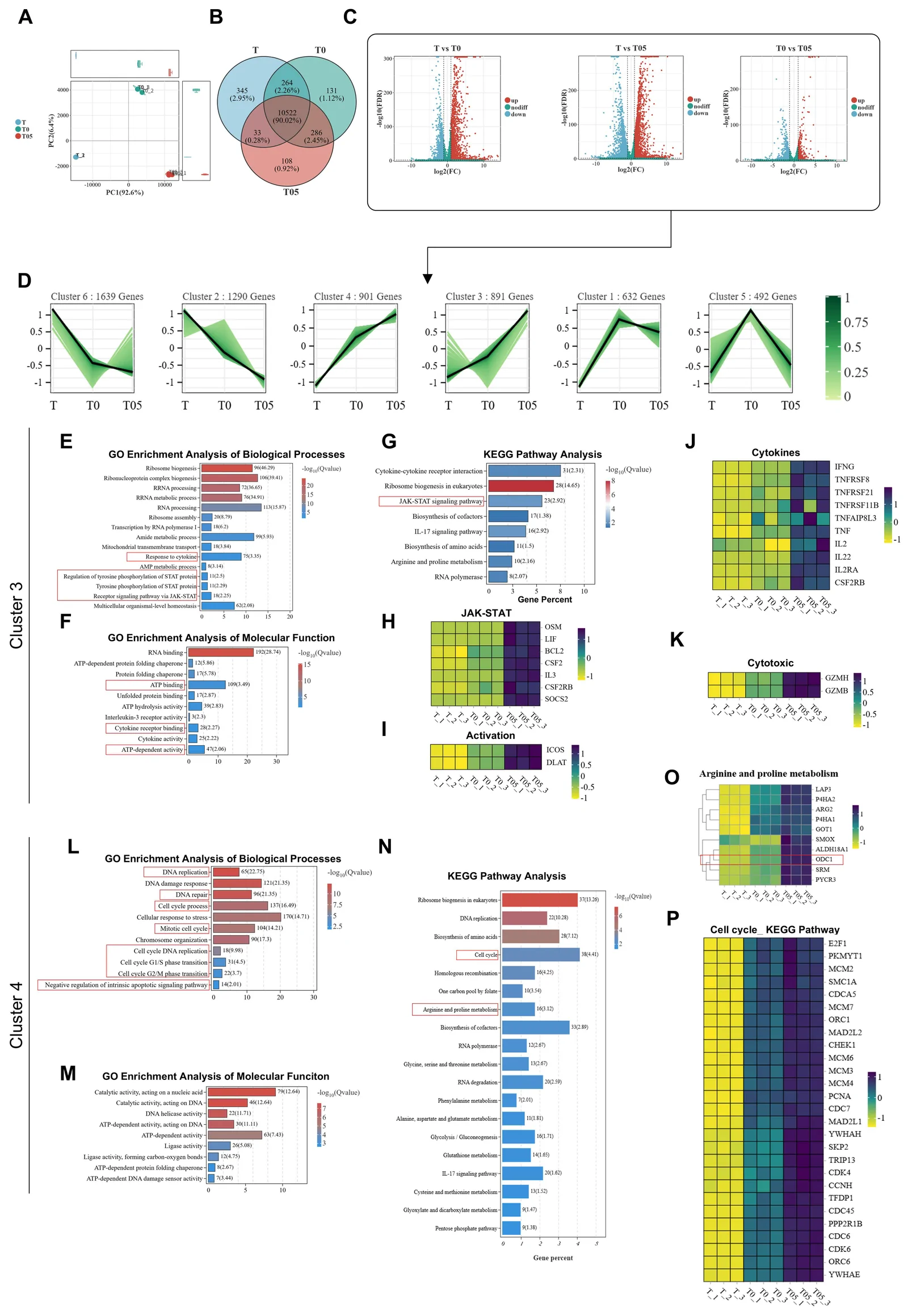
** Bulk RNA-seq analysis of the regulatory effects of the 38-mRNA vaccine on the transcriptome of CD38 CAR-T cells.** (A) Principal component analysis showing the transcriptional profile distribution of three groups: CD38 CAR-T cells cultured alone (T), CD38 CAR-T cells co-cultured with unloaded dendritic cells (T0), and CD38 CAR-T cells co-cultured with dendritic cells loaded with 0.5 μg vaccine (T05). (B) Venn diagram showing the overlap of differentially expressed genes among the three groups. (C) Volcano plots displaying differential gene expression levels in pairwise comparisons among the three groups. (D) Trend analysis of all differentially expressed genes across the three groups, identifying six distinct expression patterns (clusters). (E-F) GO enrichment analysis of biological processes and molecular functions for differentially expressed genes in Cluster 3. (G) KEGG pathway enrichment analysis of differentially expressed genes in Cluster 3. (H-K) Heatmaps showing the expression levels of JAK-STAT signaling pathway-related genes, activation-related genes, cytokine-related genes, and cytotoxicity-related genes in Cluster 3. (L-M) GO enrichment analysis of biological processes and molecular functions for differentially expressed genes in Cluster 4. (N) KEGG pathway enrichment analysis of differentially expressed genes in Cluster 4. (O-P) Heatmaps showing the expression levels of arginine and proline metabolism-related genes and cell cycle-related genes in Cluster 4. All data were derived from three independent biological replicates.

**Figure 8:**
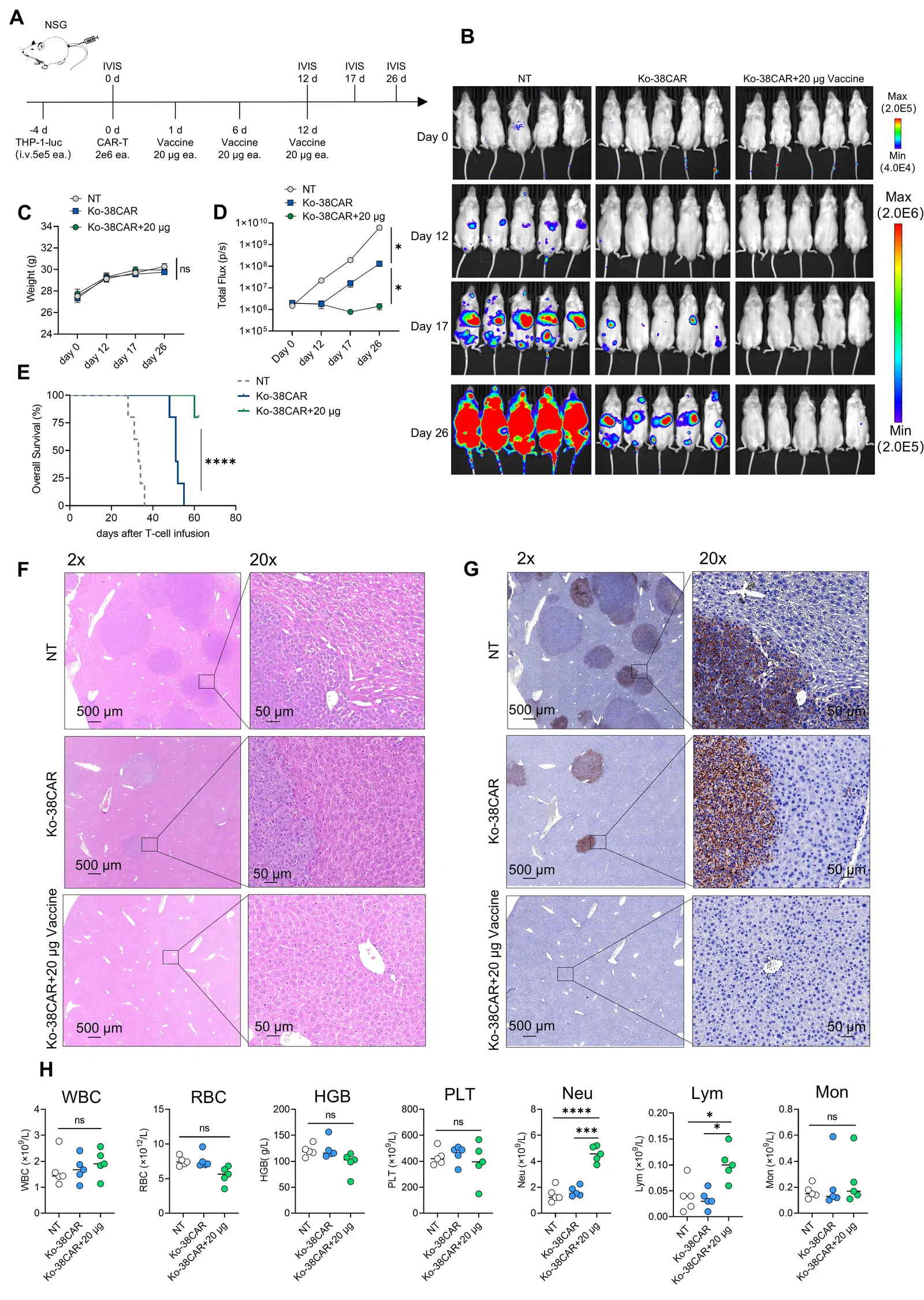
** The 38-mRNA vaccine enhances the *in vivo* antileukemic efficacy of CD38-targeted CAR-T cells.** (A) Schematic of the experimental protocol: On day -4, M-NSG mice (6–8 weeks old) were injected i.v. with luciferase-labeled THP-1 cells (5 × 10⁵/mouse) via the tail vein. On day 0, *in vivo* bioluminescence imaging was performed to assess tumor burden, and mice were randomized into groups based on tumor burden and body weight. The CAR-T treatment group and the combination treatment group received i.v. injections of 2 × 10⁶ CAR-positive CD38 CAR-T cells per mouse on day 0; the combination treatment group additionally received an i.v. injection of 20 μg/mouse 38-mRNA vaccine on day 1. Tumor burden was monitored by *in vivo* imaging on days 12, 17, and 26. (B) Representative *in vivo* bioluminescence images of tumor signals in NSG mice from each group at different time points. (C) Body weight changes of tumor-bearing mice in each group at different time points. (D) Quantitative analysis of whole-body bioluminescence intensity in NSG mice from each group at different time points (N = 5). (E) Kaplan-Meier survival curves showing overall survival of mice in different treatment groups. (F) Representative H&E-stained sections of liver tissue from each group (2× and 20×). (G) Representative CD38 immunohistochemistry-stained sections of liver tissue from each group (2× and 20×). (H) Complete blood count analysis of mice from each group (N = 5). All data are presented as mean ± SD. ns, not significant; *, *p* < 0.05; **, *p* < 0.01; ***, *p* = 0.001; ****, *p* = 0.0001.

**Figure 9:**
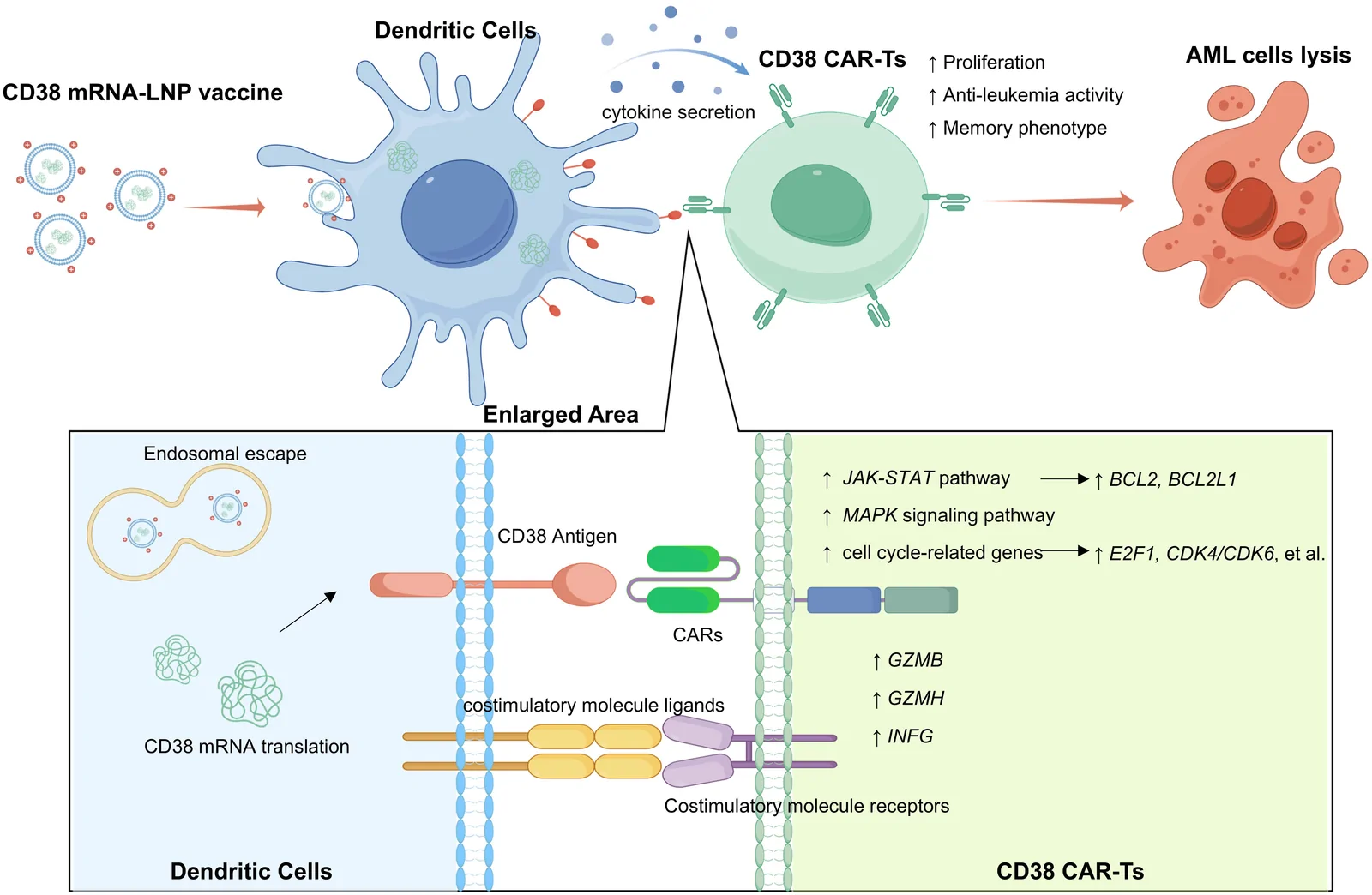
** Schematic illustration of the mechanism by which the CD38 mRNA vaccine delivers CD38 antigen to DCs, thereby activating JAK-STAT/MAPK/cell cycle-related genes in CD38 CAR-T cells to enhance antileukemic activity.** This schematic depicts the molecular mechanism through which the CD38 mRNA-LNP vaccine potentiates CD38 CAR-T cell function via DC mediation. First, the CD38 mRNA-LNP vaccine is internalized by DCs and enters endosomes. Upon endosomal disruption, mRNA encoding CD38 is released into the cytoplasm and subsequently translated by ribosomes, with the resulting CD38 protein expressed on the DC membrane. The CD38 antigen presented on the DC surface specifically binds to the CAR molecule on CD38 CAR-T cells, providing an antigen-specific activation signal. Concurrently, costimulatory molecule ligands on DCs engage costimulatory receptors on CAR-T cells, and together with multiple cytokines secreted in a paracrine manner by DCs, these signals constitute a multifaceted synergistic activation network. This signaling cascade promotes transcriptional activation of the JAK-STAT and MAPK pathways, as well as cell cycle-related genes (e.g., E2F1, CDK4/6), upregulates expression of the anti-apoptotic protein BCL2 and cytotoxic effector molecules (e.g., GZMB, GZMH), and ultimately manifests as enhanced CD38 CAR-T cell expansion, increased memory phenotype differentiation, and markedly improved antileukemic efficacy. This figure was created by Figdraw.

## Data Availability

Data are available upon reasonable request. All data generated and analyzed during this study are available from the corresponding author upon reasonable request.

## Abbreviations

AML: acute myeloid leukemia
APC: antigen-presenting cell
ATAC-seq: assay for transposase-accessible chromatin using sequencing
CAR: chimeric antigen receptor
CBA: cytometric bead array
DC: dendritic cell
IL: interleukin
JAK-STAT: Janus kinase-signal transducer and activator of transcription
Ko-38CAR: CD38-knockout CD38-targeted CAR-T cells
LNP: lipid nanoparticle
MAPK: mitogen-activated protein kinase
mRNA: messenger ribonucleic acid
ODC1: ornithine decarboxylase 1
PD-1: programmed cell death protein 1
R/R AML: relapsed/refractory acute myeloid leukemia
RNA-seq: RNA sequencing
scFv: single-chain variable fragment
T_CM_: central memory T cell
T_NAIVE_: naïve T cell
T_EM_: effector memory T cell
