# Supplementary material for: mRNA-dendritic cell antigen vaccine potentiates CD38 CAR-T Cell therapy for relapsed/refractory acute myeloid leukemia
Source: Theranostics. 2026 Aug 24;16(16):9061–79. doi: 10.7150/thno.139339 (PMC13684184; doi:10.7150/thno.139339)
Supplement: Supplementary file 1 [file thnov16p9061s1.pdf]

Supplementary Materials

Supplementary Figures S1-12

Supplementary Methods

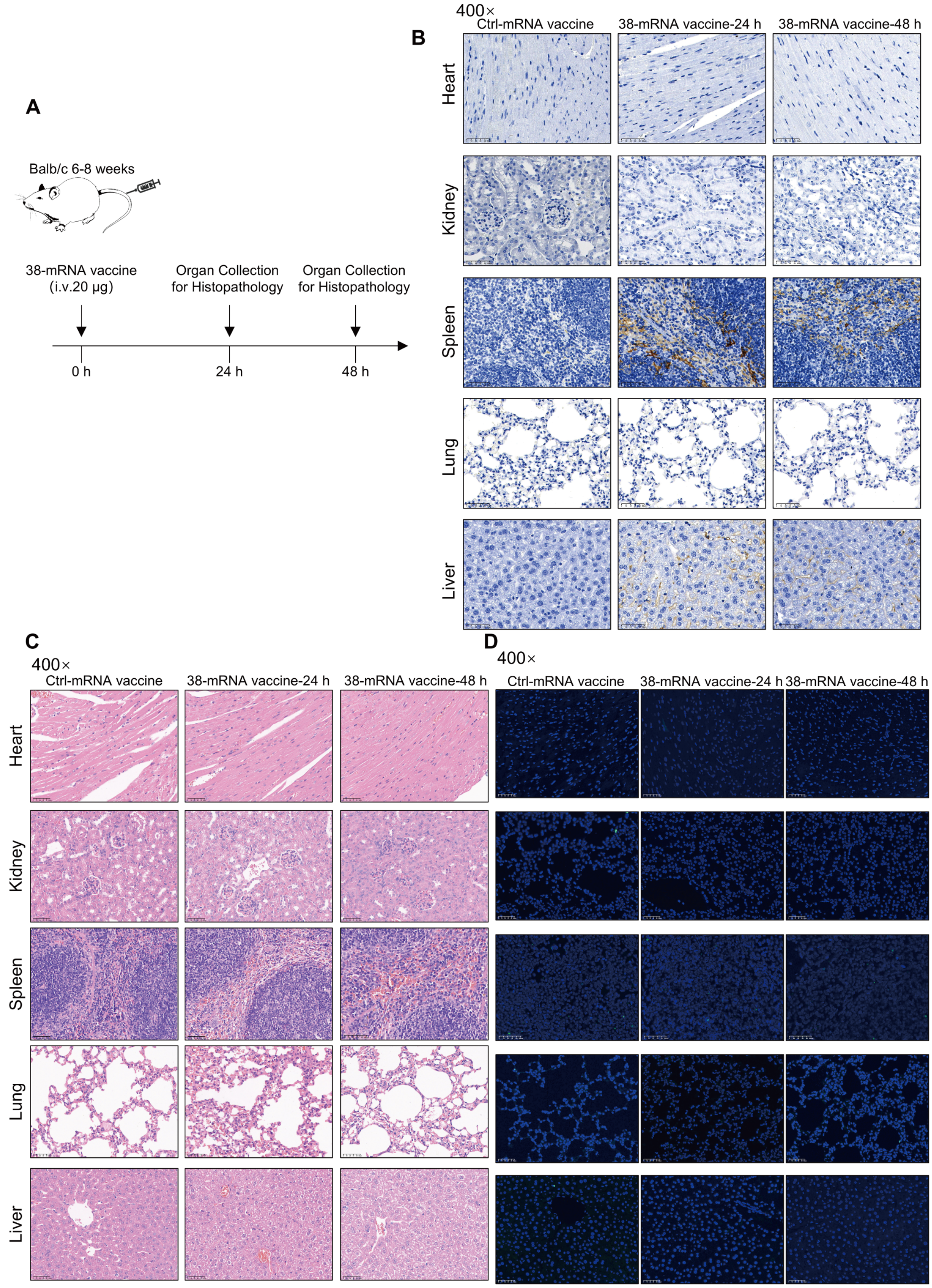

Figure S1. Histopathological analysis of in vivo distribution and safety of the 38-mRNA LNP vaccine. (A) Schematic of the experimental protocol: BALB/c mice (6–8 weeks old) were injected i.v. with 20 μg/mouse of the 38-mRNA LNP vaccine according to group assignment, while the Ctrl-

mRNA vaccine group received an equivalent volume of empty LNPs. Tissue samples were collected at 24 h and 48 h post-injection for histopathological staining. (B) CD38 immunohistochemical staining of major organs from mice in each group. (C) H&E staining of major organs from mice in each group. (D) TUNEL staining of major organs from mice in each group.

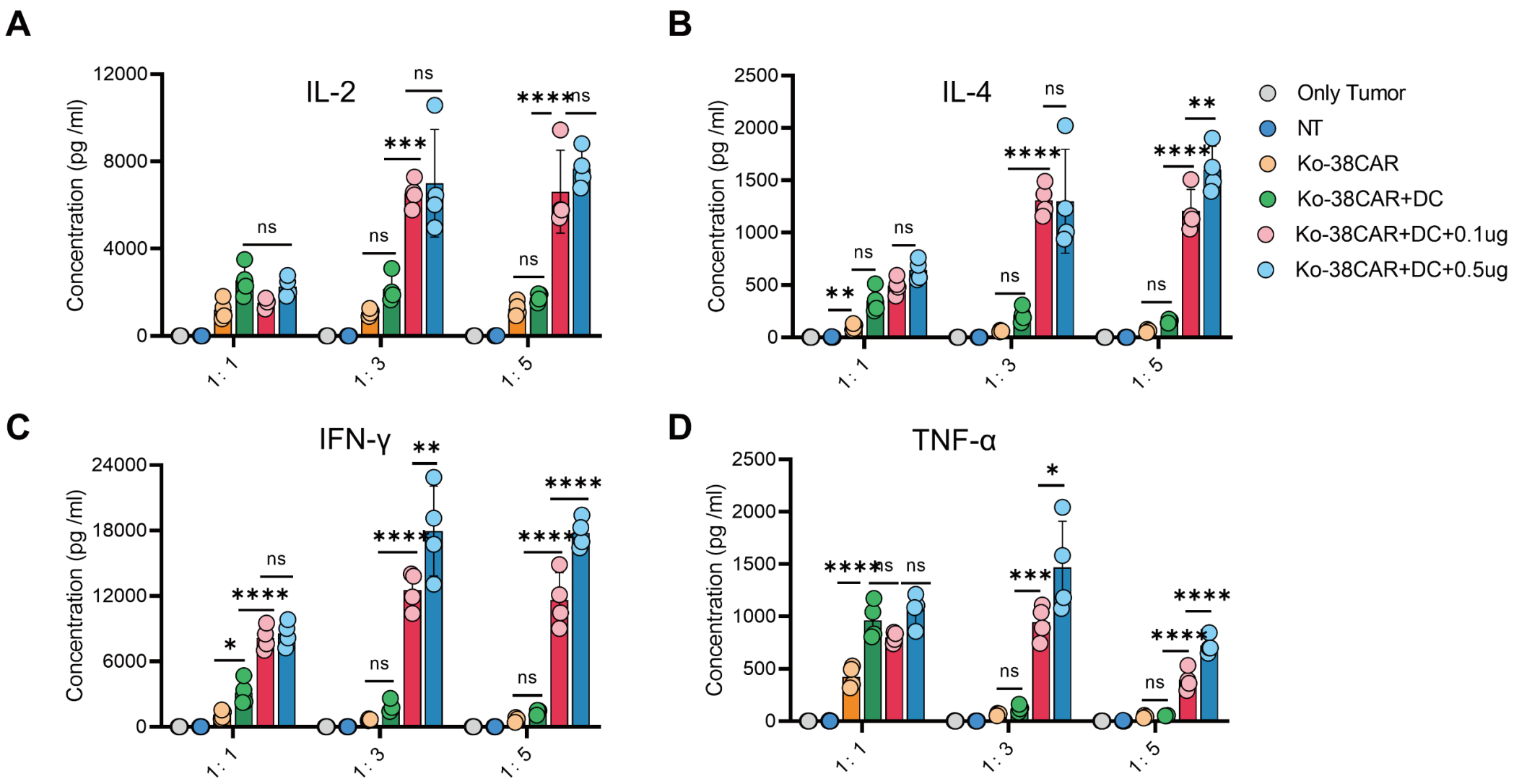

Figure S2. The 38-mRNA LNP vaccine promotes cytokine release during CD38 CAR-T cell killing. (A) CBA detection of IL-2 secretion levels in supernatants (N=4). (B) IL-4 secretion levels (N=4). (C) IFN-γ secretion levels (N=4). (D) TNF-α secretion levels (N=4). All data are presented as mean ± SD. ns, not significant; *, $p < 0.05$; **, $p < 0.01$; ***, $p = 0.001$; ****, $p = 0.0001$.

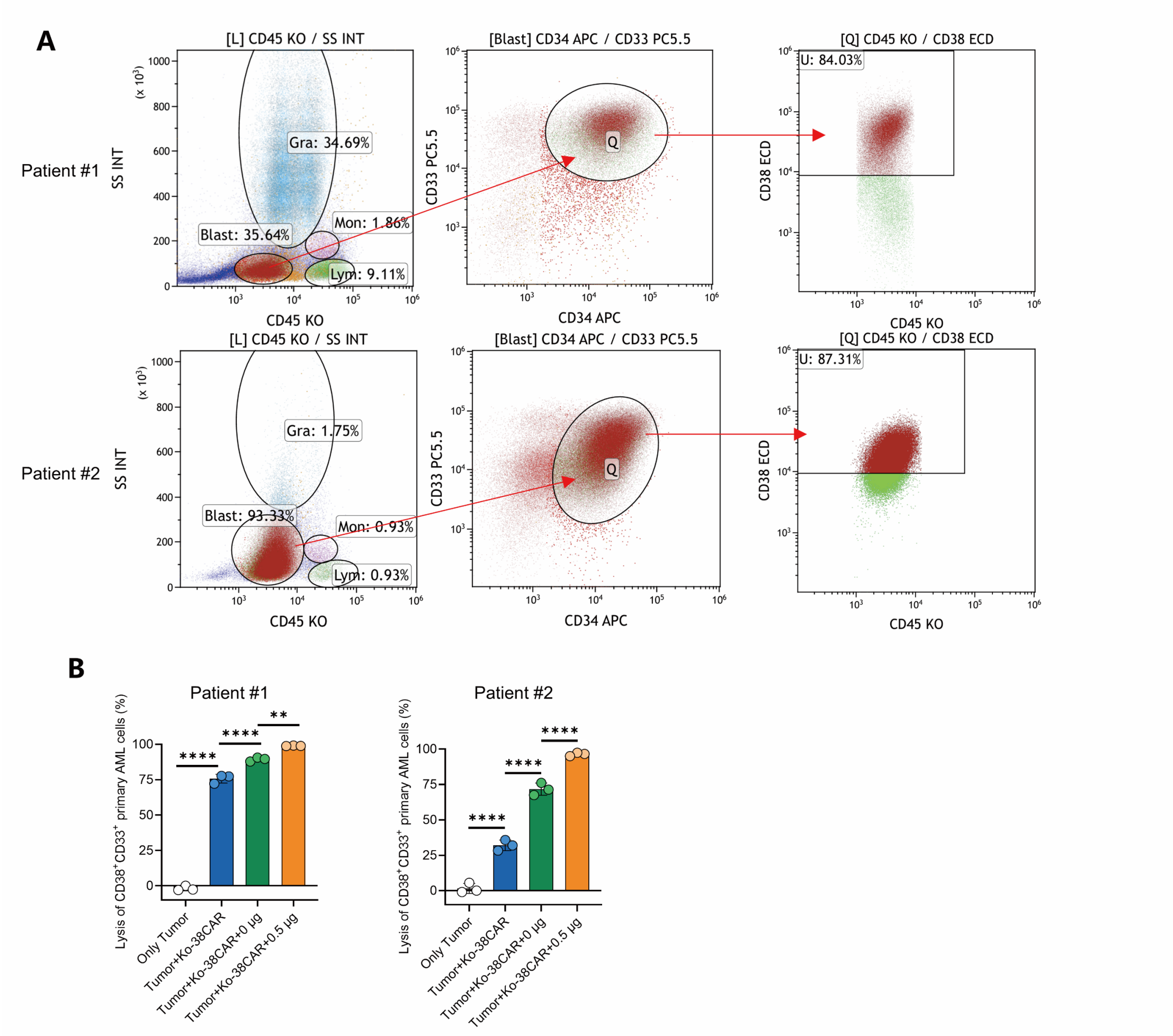

Figure S3. The 38-mRNA vaccine enhances the killing efficacy of CD38 CAR-T cells against primary CD38⁺ AML cells. (A) Flow cytometric analysis of CD38 expression on primary AML cells (gated as $CD45^{dim}CD33^{+}CD34^{+}$) from two patients with high-risk AML. (B) Bar graphs showing the cytotoxicity of CD38 CAR-T cells, pretreated with escalating doses of the CD38-mRNA vaccine, against primary AML cells derived from the two patients after 16–18 h of co-culture (N = 3). All data are presented as mean ± SD. ns, not significant; **, $p < 0.01$; ****, $p < 0.0001$.

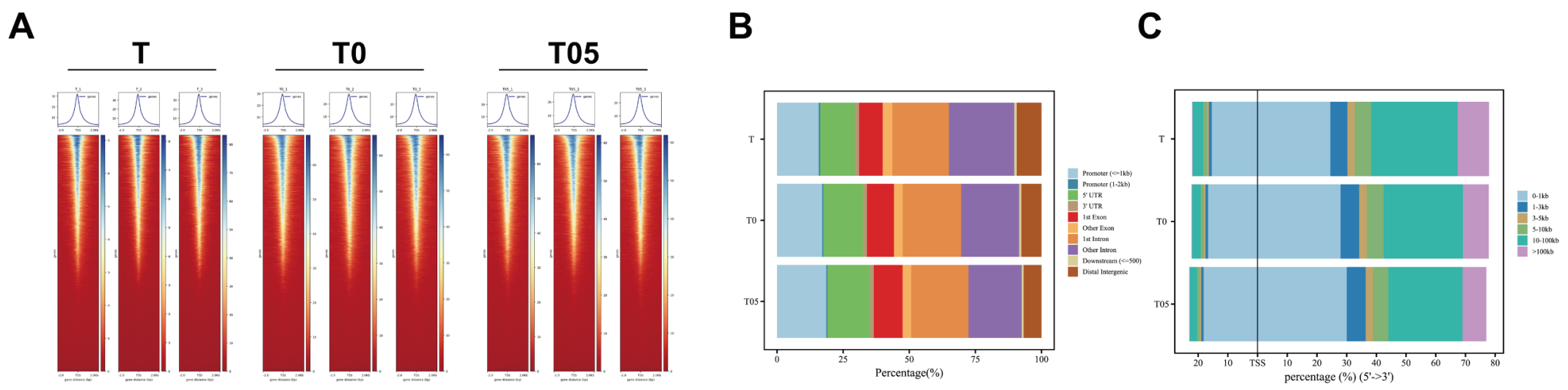

Figure S4. Overall quality assessment of ATAC-seq data and promoter region changes. (A) Enrichment of ATAC signals around transcription start sites (TSS) in each group. (B) Distribution proportions of ATAC peaks across different functional genomic elements in each group. (C) Distribution patterns of ATAC signals across gene bodies in each group.

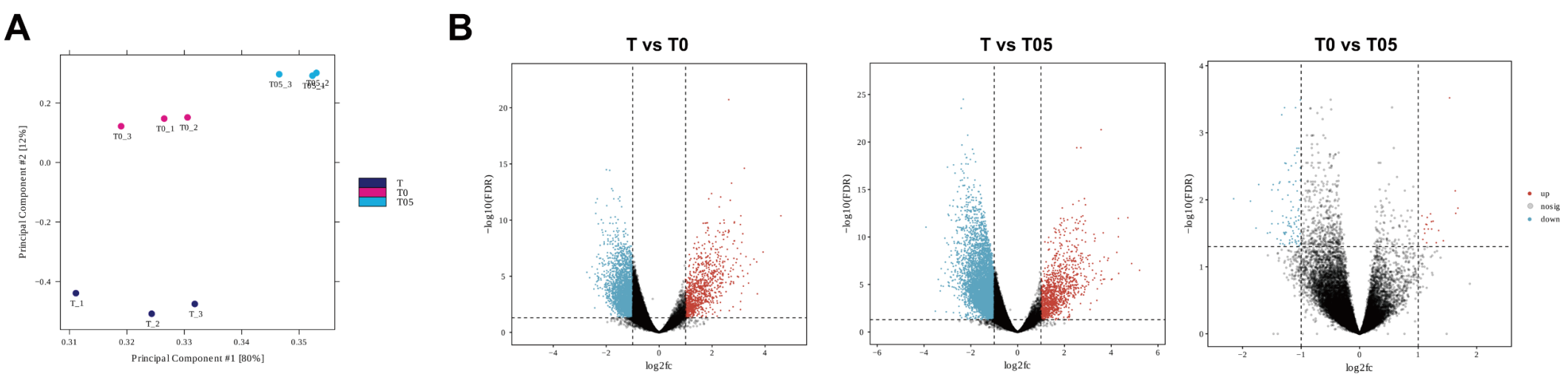

Figure S5. Principal component analysis and differential gene expression changes among different treatment groups. (A) Principal component analysis plot of different treatment groups. (B) Volcano plots of differentially expressed genes from pairwise comparisons among different groups.

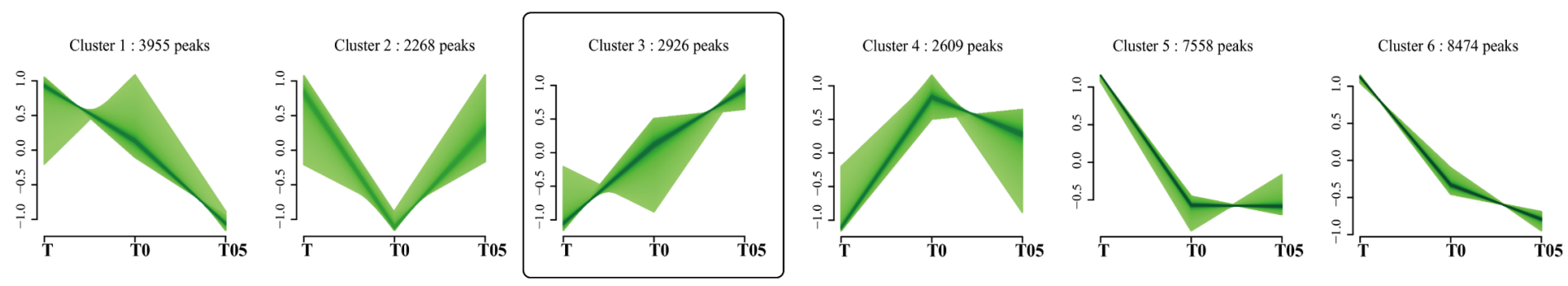

Figure S6. Trend analysis of differential chromatin accessibility peaks in CD38 CAR-T cells influenced by the 38-mRNA LNP vaccine. All differential chromatin accessibility peaks identified by ATAC-seq were subjected to trend analysis and clustered into six distinct clusters.

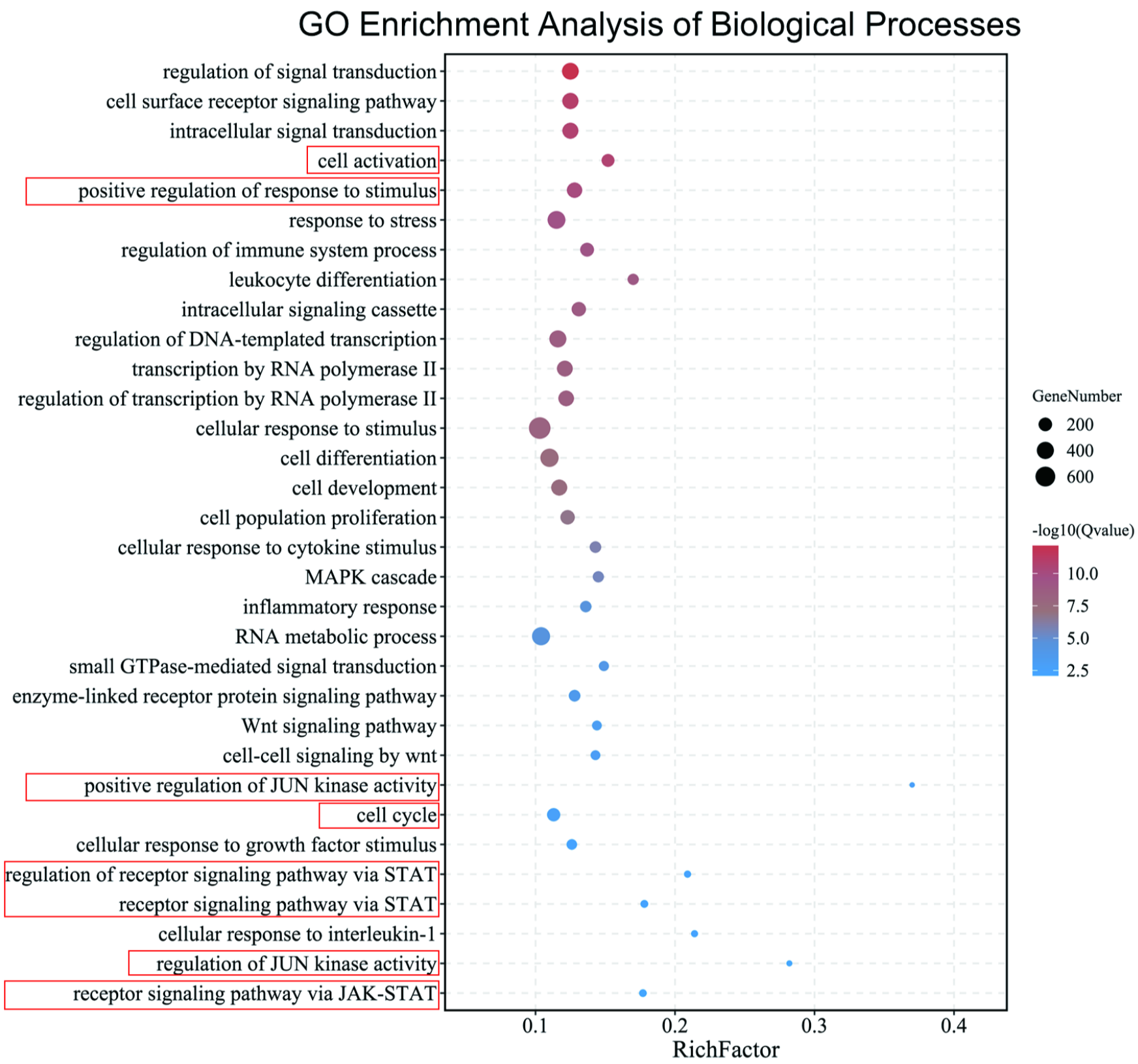

Figure S7. GO pathway enrichment analysis of the upregulated gene cluster (Cluster 3). GO pathway enrichment analysis was performed on the gene cluster exhibiting an upward trend (Cluster 3) identified in the trend analysis, and the results are presented as a bubble plot.

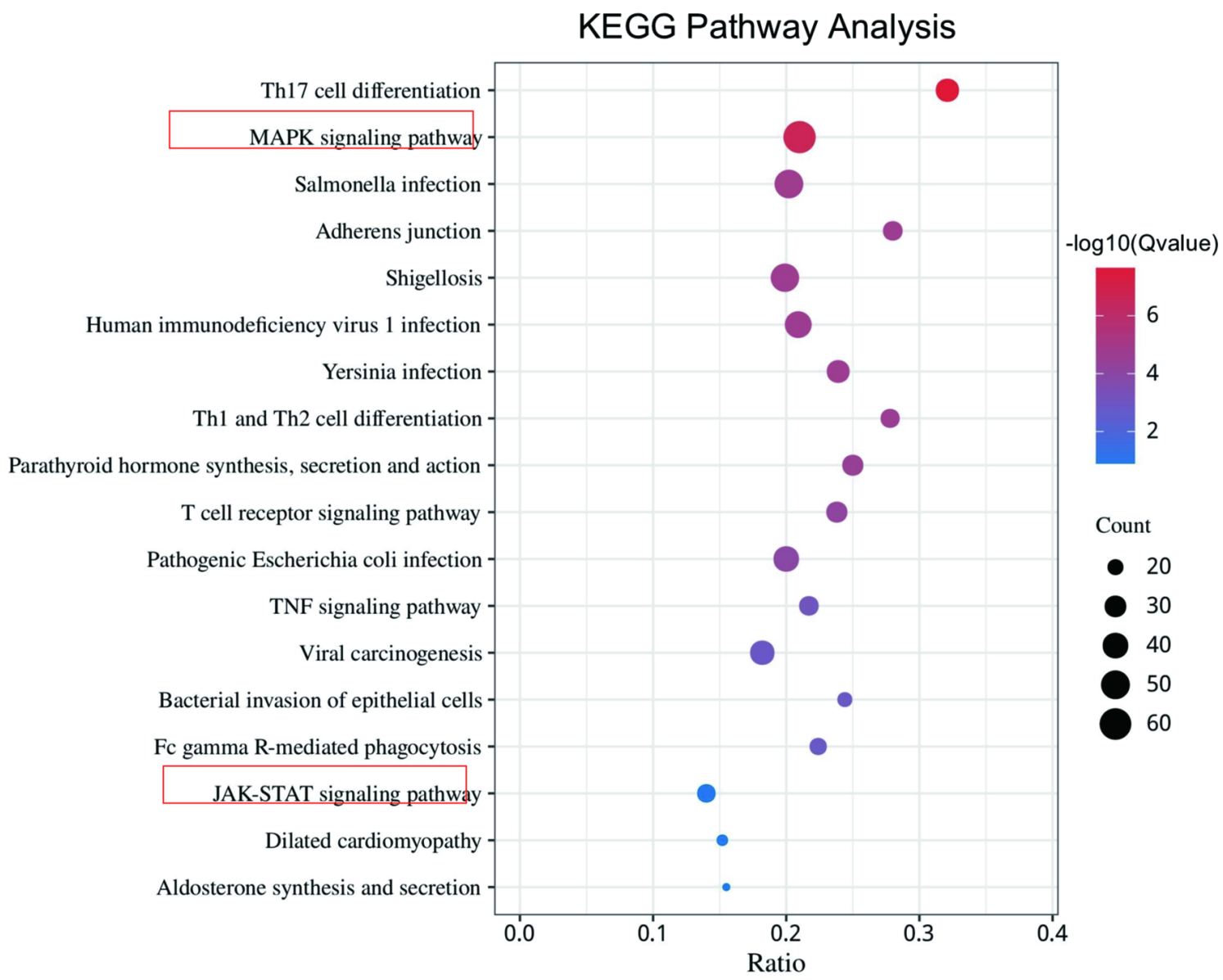

Figure S8. KEGG pathway enrichment analysis of the upregulated gene cluster (Cluster 3).

KEGG pathway enrichment analysis was performed on the gene cluster exhibiting an upward trend (Cluster 3) identified in the trend analysis, and the results are presented as a bubble plot.

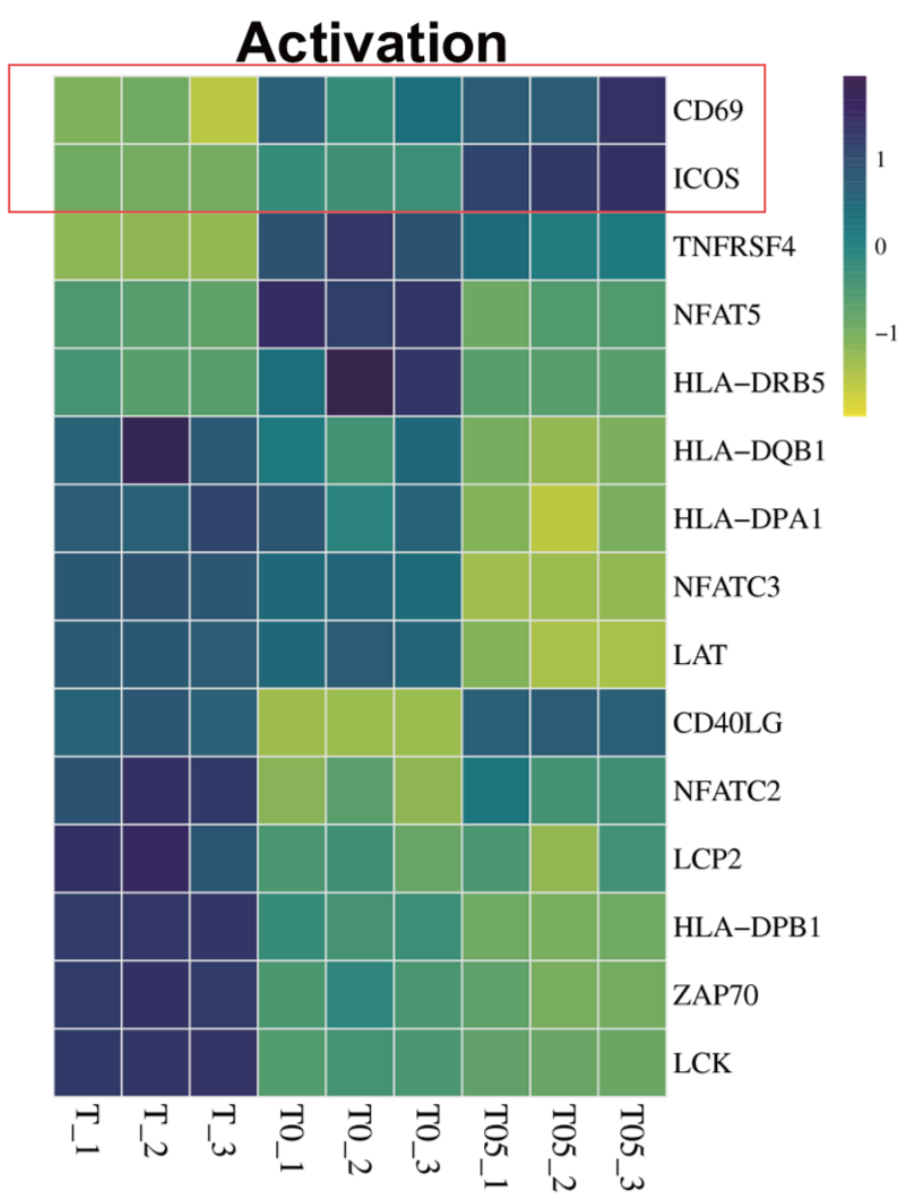

Figure S9. Heatmap analysis of activation-related gene expression profiles. Expression levels of activation-related genes within the continuously upregulated gene cluster (Cluster 3) identified

in the trend analysis were analyzed and presented as a gene expression heatmap.

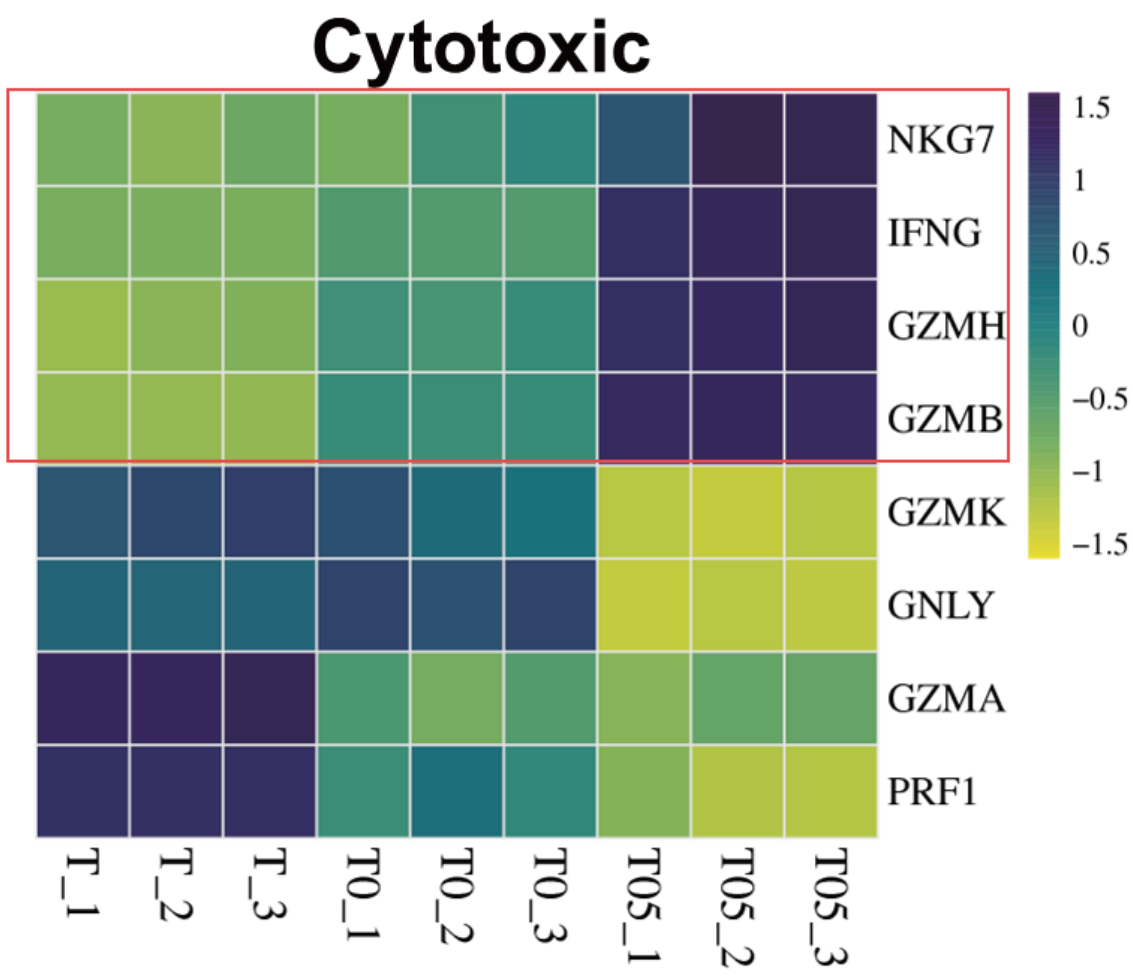

Figure S10. Heatmap analysis of cytotoxicity-related gene expression profiles. Expression levels of cytotoxicity-related genes within the continuously upregulated gene cluster (Cluster 3) identified in the trend analysis were analyzed and presented as a gene expression heatmap.

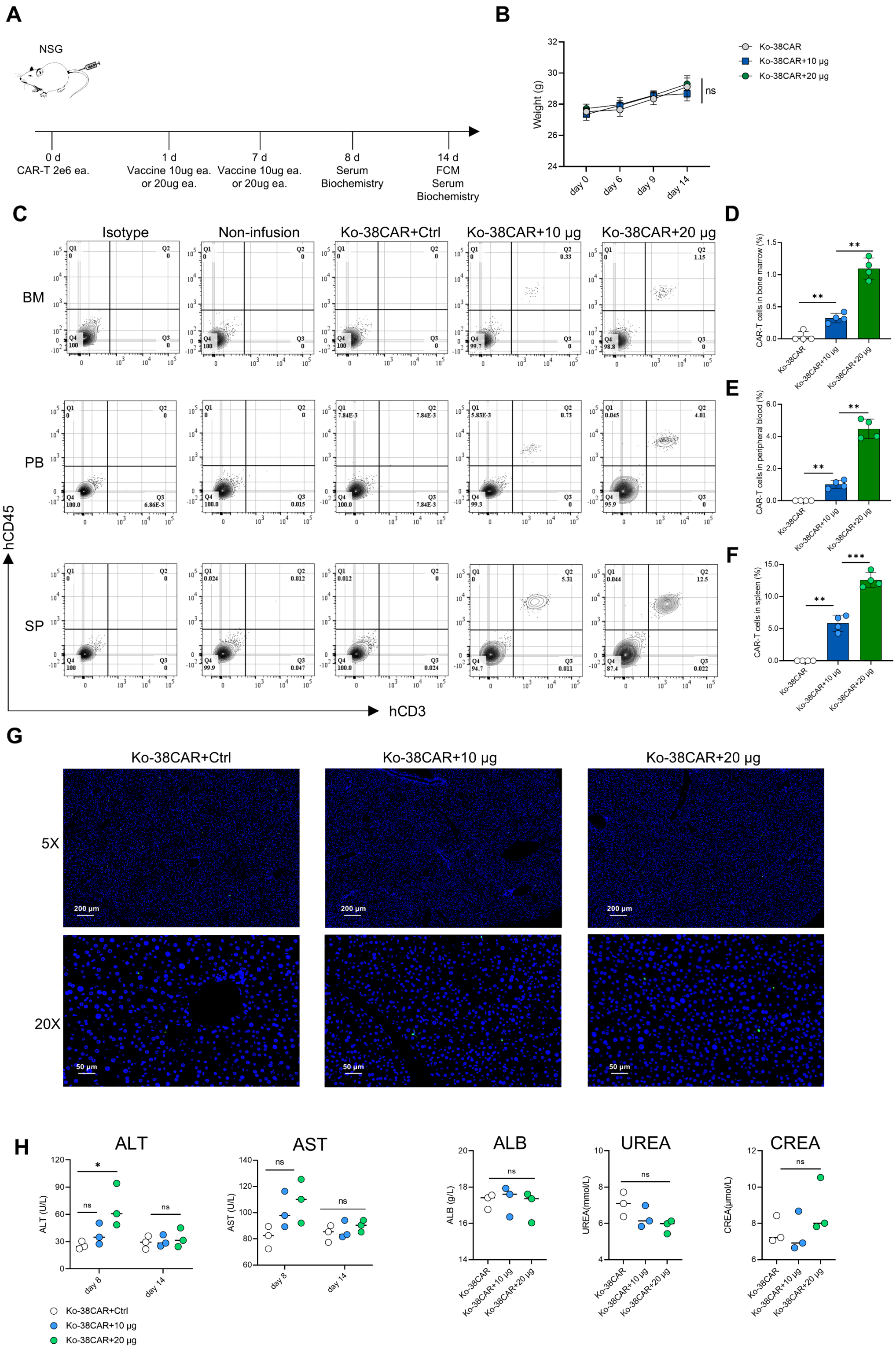

Figure S11. In vivo proliferation and safety evaluation of CD38-targeted CAR-T cells promoted by the 38-mRNA LNP vaccine. (A) Schematic of the experimental protocol: On day 0, M-NSG mice (6–8 weeks old) were injected i.v. with 2×10^6 CAR-positive CD38 CAR-T cells.

On day 1, mice received either 10 μg or 20 μg of the 38-mRNA LNP vaccine per mouse according to group assignment, while the control group received an equivalent volume of empty LNPs as the 20 μg vaccine group. A second dose was administered on day 7, blood biochemistry was assessed on day 8, and mice were sacrificed on day 14, with tissues collected for flow cytometry, blood biochemistry analysis, and TUNEL staining. (B) Body weight changes in each group during the experimental period. (C–F) Flow cytometric detection of CAR-T cell ($CD3^{+}CD45^{+}$) proportions in bone marrow, spleen, and peripheral blood. Isotype control and non-infused groups were set as negative controls (N=4). (G) Representative TUNEL staining images of liver tissues (5× and 20×) from each group, used to evaluate the distribution of apoptotic cells in liver tissues across different treatment groups; scale bars = 200 μm (5×) and 50 μm (20×). (H) Serum biochemistry analysis, including ALT, AST, ALB, UREA, and CREA (N=3). All data are presented as mean ± SD. ns, not significant; **, $p < 0.01$; ***, $p < 0.001$.

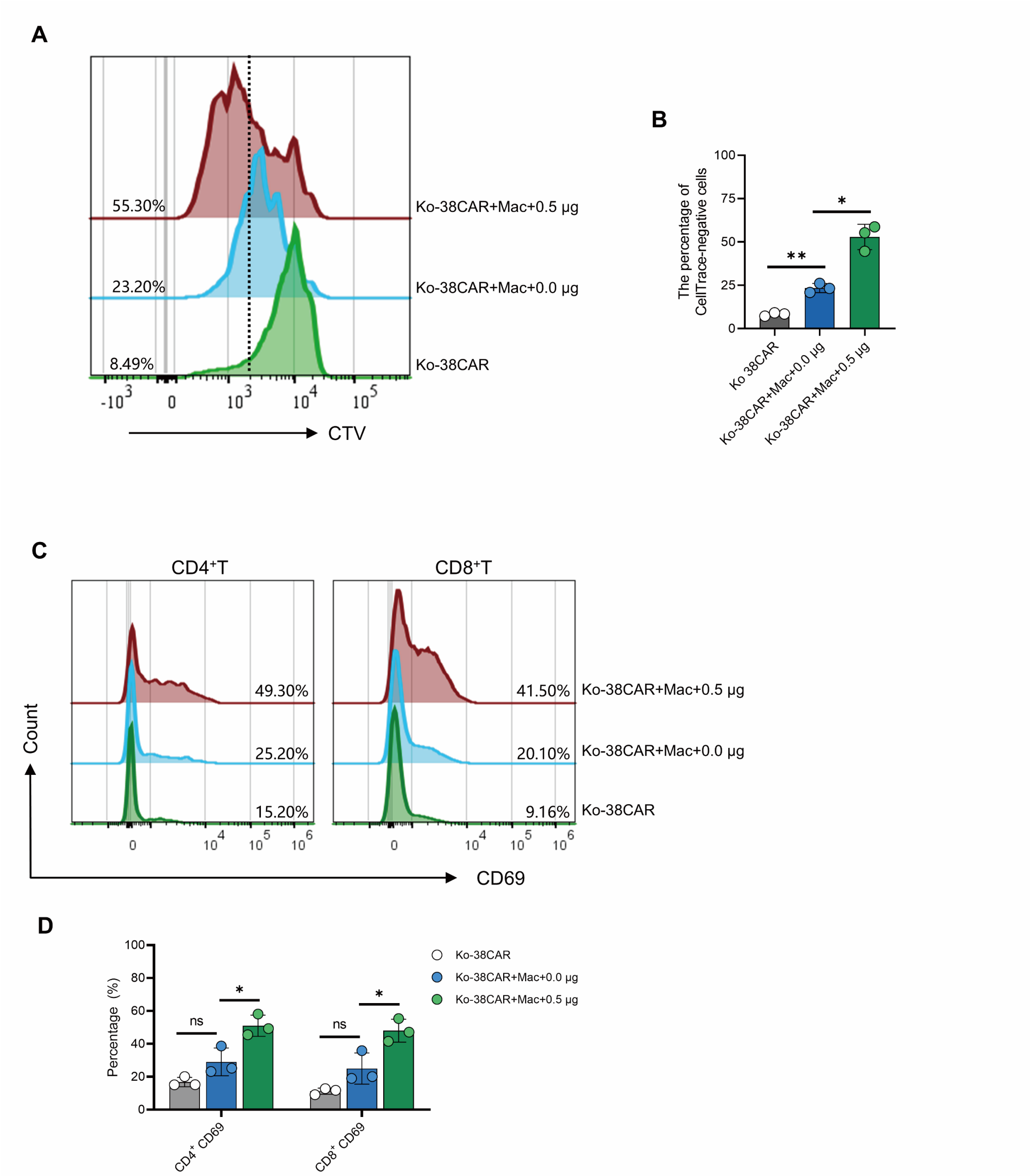

Figure S12. The 38-mRNA vaccine-transfected macrophages promote the activation and proliferation of CD38 CAR-T cells. (A–B) Representative flow cytometry plots and bar graph showing the proliferation of CD38 CAR-T cells after 96 h of co-culture with macrophages transfected with the 38-mRNA vaccine (N=3). (C–D) Representative flow cytometry plots and bar graph showing the activation of CD38 CAR-T cells after 24 h of co-culture with macrophages transfected with the 38-mRNA vaccine (N = 3). Mac, macrophages. All data are presented as mean ± SD. ns, not significant; *, $p < 0.05$; **, $p < 0.01$.

Supplementary Methods

Macrophage differentiation and culture

For macrophage differentiation, with reference to previously reported literature [1], monocytes were cultured in RPMI 1640 GlutaMAX™ supplemented with 50 IU/mL penicillin, 50 μg/mL streptomycin, 1 mM sodium pyruvate, non-essential amino acids, and 10% fetal bovine serum (10099141C, GIBCO), together with 1,000 IU/mL recombinant human GM-CSF (91113ES60; Yeasen, Shanghai, China) for 5–7 days, and then harvested for subsequent use.

Construction of chimeric antigen receptors (CAR) and lentiviral packaging

The design of the chimeric antigen receptor (CAR) construct and the packaging of the lentiviral vectors were performed by GenChem Inc. The single-chain variable fragment (scFv) was derived from Daratumumab. The intracellular signaling domain comprised the co-stimulatory molecule 4-1BB and the CD3ζ signaling module. The CAR expression was driven by the elongation factor-1 alpha (EF-1α) promoter. An enhanced green fluorescent protein (EGFP) reporter gene was linked to the CAR sequence via a self-cleaving P2A peptide. This entire expression cassette was cloned into a second-generation CAR backbone plasmid. For lentivirus production, the CAR transfer plasmid was co-transfected with the packaging plasmids psPax2 and pMD2.G into HEK-293T cells using standard protocols. The viral supernatant was collected approximately 48 hours post-transfection. The lentiviral particles were then concentrated by ultracentrifugation at 120,000 × g for 2 hours. The concentrated viral pellets were resuspended in DPBS, aliquoted, and stored at -80°C for subsequent use.

Isolation, Expansion, CD38-KO and lentiviral transduction of human T cells

T cells were isolated from peripheral blood samples of healthy donors. Briefly, peripheral blood

mononuclear cells (PBMCs) were first separated by Ficoll density gradient centrifugation. T cells were then enriched from PBMCs using CD3-positive selection magnetic beads (Miltenyi Biotec). The enriched T cells were activated with CD3/CD28/CD2 T cell activator beads (Miltenyi Biotec) and cultured in X-VIVO medium (Lonza) supplemented with 10% fetal bovine serum (FBS) and 100IU/mL recombinant human interleukin-2 (PeproTech). During the culture period, the medium was refreshed every two days, and the cell seeding density was maintained at 1×10^6 cells per milliliter.

The CD38 knockout method and sgRNA sequences were based on previously reported literature[2]. To knock out endogenous CD38 in CD38 CAR-T cells, CRISPR-Cas9 ribonucleoprotein electroporation was employed. Briefly, T cells were harvested 48 hours post-activation and resuspended in electroporation buffer. Cas9 protein (GenScript) was mixed with CD38-targeting sgRNA (GenScript) at a 1:2 molar ratio and incubated at room temperature for 15 minutes to form RNP complexes. The RNP complexes were added to the cell suspension, and electroporation was performed using an electroporator. Following electroporation, cells were transferred to complete medium supplemented with IL-2 and cultured for an additional 72 hours. Subsequently, cells were transduced with lentivirus at a multiplicity of infection of 1, and the virus-cell mixture was centrifuged at 600 × g for 1 hour to enhance infection efficiency, followed by overnight incubation. The medium was replaced with fresh complete medium the next day.

Measurement of pH, particle size, and encapsulation efficiency of the 38-mRNA vaccine

As previously described, pH measurements were performed using a Seven Compact S210 instrument (Mettler Toledo, Switzerland). Particle size of the LNPs was assessed by dynamic light scattering using a Zetasizer Nano-ZS (Malvern). mRNA encapsulation efficiency was determined using the Quant-it RiboGreen RNA assay (Life Technologies). The procedure involved incubation of samples at 37°C for

10 minutes in the presence or absence of 1% Triton X-100 (Sigma-Aldrich). Following the addition of RiboGreen reagent, fluorescence intensity (excitation/emission: 480/520 nm) was recorded for untreated samples (representing unencapsulated mRNA) and Triton X-100-treated samples (representing total mRNA). Encapsulation efficiency was calculated as: (total mRNA - unencapsulated mRNA) / total mRNA × 100%.

Cryo-transmission electron microscopy imaging

Cryo-transmission electron microscopy images were acquired using an FEI Talos F200C transmission electron microscope (Thermo Fisher Scientific). Sample preparation was performed using an FEI Vitrobot at 4°C and 100% relative humidity. Prior to sample loading, copper TEM grids were subjected to a 90-second plasma etching process. Subsequently, 3.5 μL of the LNP solution was pipetted onto the grid within the sample chamber. Using an automated system, blotting was performed for 5 seconds, after which the sample was rapidly plunged into liquid ethane for vitrification. The vitrified samples were then transferred to a Gatan 626 cryo-holder and observed using the FEI Talos F200C microscope operated at an accelerating voltage of 200 kV, equipped with an FEI Ceta camera. Image acquisition was performed using EPU and SerialEM software.

Animals

All animal experiments were approved by the Institutional Animal Care and Use Committee of Soochow University (Suzhou, China). All mice were housed in the animal facility of Soochow University under a 12-hour light/dark cycle, with ambient temperature maintained at 20–22°C and humidity at 40–70%. BALB/c mice and NOD.Cg-PrkdcscidIl2rgem1Smoc (M-NSG) mice (6–8 weeks old, male) were purchased from Shanghai Model Organisms Center, Inc.

In vivo biodistribution of mRNA vaccines

BALB/c mice (6–8 weeks old) were injected intravenously via the tail vein with Luciferase-mRNA vaccine (0.5 mg/kg), 38-mRNA vaccine (0.5 mg/kg), or empty control vaccine. For mice administered the 38-mRNA vaccine, animals were sacrificed at 24 h and 48 h post-injection, and organs and bone marrow were collected for further analysis, including flow cytometry and histopathological examination. For mice administered the Luciferase-mRNA vaccine, organ distribution was assessed at designated time points using the IVIS Lumina Series III in vivo imaging system following intraperitoneal injection of 150 mg/kg D-Luciferin Potassium Salt (dd1210, Vazyme Biotech Co.,Ltd) under isoflurane anesthesia. Image analysis was performed using Living Image software.

Adoptive cell transfer and In vivo expansion study of the 38-mRNA vaccine

With reference to previous literature[3], CD38-targeted CAR-T cells were infused intravenously into M-NSG mice at a dose of 2×10^6 CAR^+ cells per mouse. Subsequently, mice were randomly assigned to the empty control vaccine group, the low-dose 38-mRNA vaccine group (0.5 mg/kg), or the high-dose 38-mRNA vaccine group (1 mg/kg). The 38-mRNA vaccine was administered on days 1 and 6 according to the assigned dosage. Mice were sacrificed 7 days after the second treatment, and peripheral blood, spleen, and bone marrow were collected for flow cytometric analysis, serum biochemistry, and complete blood count evaluation.

In vivo xenograft mouse models

With reference to previous literature, a systemic disseminated AML model was established by intravenous injection of AML cells. M-NSG mice were inoculated with 5×10^5 THP-1-luc tumor cells per mouse via the tail vein. On day 4 post-inoculation, mice were imaged and weighed, and then randomly assigned to three groups based on tumor burden and body weight: a non-transduced T cell control group (matched for cell number with the CAR^+ group), a Ko-38CAR group (CAR^+ cells at 2×10^6

per mouse), and a Ko-38CAR+38-mRNA vaccine group (CAR^+ cells at 2×10^6 per mouse+20 μg vaccine per mouse). Subsequently, tumor progression was assessed dynamically using the IVIS Lumina Series III in vivo imaging system as previously described. Mice were sacrificed at designated time points, and peripheral blood and tissue samples were collected for complete blood count and histopathological analysis.

H&E staining, TUNEL assay, and immunohistochemistry

According to previously reported protocols, tissue samples were fixed in formalin, embedded in paraffin, and sectioned at 3–4 μm thickness for subsequent analyses.

H&E staining was performed according to the manufacturer's instructions (Solarbio). Briefly, paraffin sections were deparaffinized in xylene and rehydrated through a graded ethanol series. Nuclei were stained with hematoxylin solution, followed by differentiation and bluing steps. Cytoplasm and extracellular matrix were counterstained with eosin solution. Subsequently, sections were dehydrated through a graded ethanol series, cleared in xylene, mounted with neutral gum, and observed under a light microscope.

TUNEL staining was performed according to the manufacturer's instructions (Solarbio, Beijing, China). Briefly, tissue sections were deparaffinized and rehydrated, followed by antigen retrieval with proteinase K to increase membrane permeability. Sections were then incubated with TUNEL reaction mixture for 60 minutes at 37°C in the dark. After washing, sections were counterstained with DAPI (C0065, Solarbio, Beijing, China) and observed under a fluorescence microscope.

Immunohistochemistry, deparaffinized and rehydrated tissue sections were subjected to antigen retrieval (heat-induced epitope retrieval with citrate buffer), followed by blocking of endogenous peroxidase activity and blocking of non-specific binding sites with serum or BSA. Sections were incubated with

anti-human CD38 primary antibody (Abcam) overnight at 4°C. After washing with PBS, sections were incubated with horseradish peroxidase-conjugated goat anti-rabbit IgG secondary antibody (Cell Signaling Technology) for 30–60 minutes at room temperature. Antigen localization was visualized by DAB chromogenic reaction, and nuclei were counterstained with hematoxylin before mounting and observation.

Complete blood count and serum biochemistry analysis in mice

Experimental mice were anesthetized with isoflurane, and peripheral blood was collected via the orbital venous plexus. The collected blood was immediately transferred into anticoagulant tubes containing mouse-ACD and gently mixed. After thorough mixing, anticoagulated whole blood samples were analyzed using an automated hematology analyzer (Beckman Coulter) for complete blood count analysis. An additional aliquot of whole blood was centrifuged to separate serum, and serum biochemistry parameters were measured using an automated biochemistry analyzer (Beckman Coulter).

Bulk RNA-Seq, ATAC-Seq, and Data analysis

Bulk RNA-seq

GFP-positive CAR-T cells co-cultured with dendritic cells were sorted by flow cytometry and submitted to Gene Denovo Biotechnology Co., Ltd. for bulk RNA-seq. Briefly, total RNA was extracted from samples, and RNA concentration, purity, and integrity were assessed using a NanoDrop spectrophotometer and an Agilent 2100 Bioanalyzer. Subsequently, library construction was performed using magnetic bead purification technology, including mRNA capture, fragmentation, cDNA synthesis, end repair, and adapter ligation. The constructed libraries were subjected to high-throughput sequencing. After sequencing, bioinformatics analysis was performed for quality control, sequence alignment, and gene expression quantification to identify gene expression profiles and differentially expressed genes.

ATAC-seq

GFP-positive CAR-T cells co-cultured with dendritic cells were sorted by flow cytometry and submitted to Gene Denovo Biotechnology Co., Ltd. for ATAC-seq. The experimental procedure was as follows: nuclei were extracted and treated with hypotonic lysis buffer to release intact nuclei. The Tn5 transposase was used to cleave open chromatin regions and insert sequencing adapters. Adapter-ligated DNA fragments were PCR-amplified to increase library complexity. The amplified libraries were purified and quantified, followed by sequencing on the Illumina high-throughput sequencing platform. After sequencing, bioinformatics analysis was performed for quality control, sequence alignment, and peak calling to identify open chromatin regions, transcription factor binding sites, and nucleosome positioning features.

Proliferation assay

CAR-transduced human T cells were labeled with 2.5 μM CellTrace Violet (CTV, Thermo Fisher Scientific) and co-cultured with 38-mRNA vaccine-transfected dendritic cells at an effector-to-DCs or macrophages ratio of 10:1 in 96-well plates. After 96 hours of co-culture, T cell proliferation was assessed by flow cytometry.

Multiplex cytokine analysis

The BD™ Cytometric Bead Array (CBA) was used to quantify multiple cytokines in the following samples: culture supernatants of dendritic cells transfected with the 38-mRNA vaccine, supernatants from co-cultures of transfected dendritic cells and CAR-T cells, and supernatants from cytotoxicity assays. Cell culture supernatants were collected and diluted to an appropriate concentration range. Subsequently, the assay was performed according to the manufacturer's instructions, including

establishment of standard curves, incubation of supernatants with capture beads, and detection by flow cytometry. Data were analyzed using CBA Analysis Software.

Cytotoxicity assay

To evaluate the killing capacity of CAR-T cells against tumor cells, THP-1 and MOLM-13 target cells were labeled with CellTrace Violet (CTV, Thermo Fisher Scientific) to distinguish them from CAR-T cells. Labeled target cells were co-cultured with CAR-T cells or non-transduced T cells at effector-to-target ratios of 1:1, 1:3, and 1:5 for 16–18 hours. Cells and supernatants from each well were collected and stained using an Annexin V and propidium iodide (PI) apoptosis detection kit (E-CK-A217, Elabscience Biotechnology Co.,Ltd). Data were analyzed using FlowJo v10.1 software.

Bone marrow samples from two patients with high-risk AML were subjected to red blood cell lysis and subsequently stained with fluorescence-conjugated antibodies against CD45-KO, CD34-APC, CD33-PerCP-Cy5.5, and CD38-ECD (all purchased from Beckman Coulter). Stained cells were acquired on a flow cytometer (Beckman Coulter) and analyzed using Kaluza flow cytometry analysis software. AML blasts were identified by gating on the $CD45^{dim}CD33^{+}CD34^{+}$population, and CD38 expression levels were evaluated accordingly. Following the method described in a previous report [4], bone marrow mononuclear cells were isolated from two AML patients and labeled with CellTrace Violet (CTV; Thermo Fisher Scientific) to distinguish them from CAR-T cells. The labeled target cells were then co-cultured with CAR-T cells generated from three different healthy donors or with non-transduced T cells at an effector-to-target ratio of 1:2 for 16–18 hours. Cells from each well were collected, stained with CD33-APC, CD38-FITC, and PI (Biolegend), and subsequently analyzed by flow cytometry (BD bioscience). Data were analyzed using FlowJo v10.1 software. Patient information is as follows: Patient #1, male, 18 years old, AML-M4, karyotype 47, XY, +8[1]/49, idem, +21, +22[2]/46, XY[17], FLT3-

ITD positive, ASXL1, EP300, and FAT1 mutations, high risk; Patient #2, female, 57 years old, AML, karyotype 44, XX, -2, -5, -7, -9, i(17)(q10), +m1, +m2[10], TP53 positive, high risk.

References

1. Zuo S, Wang Y, Bao H, Zhang Z, Yang N, Jia M, et al. Lipid synthesis, triggered by PPARγ T166 dephosphorylation, sustains reparative function of macrophages during tissue repair. Nat Commun. 2024; 15: 7269.
2. Liao C, Wang Y, Huang Y, Duan Y, Liang Y, Chen J, et al. CD38-Specific CAR Integrated into CD38 Locus Driven by Different Promoters Causes Distinct Antitumor Activities of T and NK Cells. Adv Sci (Weinh). 2023; 10: e2207394.
3. Reinhard K, Rengstl B, Oehm P, Michel K, Billmeier A, Hayduk N, et al. An RNA vaccine drives expansion and efficacy of claudin-CAR-T cells against solid tumors. Science. 2020; 367: 446-53.
4. Gomes-Silva D, Atilla E, Atilla PA, Mo F, Tashiro H, Srinivasan M, et al. CD7 CAR T Cells for the Therapy of Acute Myeloid Leukemia. Mol Ther. 2019; 27: 272-80.
